# Smoking and coronary artery disease risk in patients with diabetes: A Mendelian randomization study

**DOI:** 10.3389/fimmu.2023.891947

**Published:** 2023-01-26

**Authors:** Songzan Chen, Fangkun Yang, Tian Xu, Yao Wang, Kaijie Zhang, Guosheng Fu, Wenbin Zhang

**Affiliations:** ^1^ Department of Cardiology, Sir Run Run Shaw Hospital, School of Medicine, Zhejiang University, Hangzhou, China; ^2^ Key Laboratory of Cardiovascular Intervention and Regenerative Medicine of Zhejiang Province, Hangzhou, China; ^3^ Department of Cardiology, Ningbo First Hospital, Ningbo, China

**Keywords:** mendelian randomization, coronary artery disease, cardiovascular risk factors, smoking, diabetes

## Abstract

**Background:**

Previous observational studies have shown an association between smoking and coronary artery disease (CAD) in patients with diabetes. Whether this association reflects causality remains unestablished. This study aimed to explore the causal effect of smoking on CAD in patients with diabetes.

**Methods:**

Genetic signatures for smoking were extracted from a large genome-wide association study (GWAS), consisted of up to 1.2 million participants. Four smoking phenotypes were included: smoking initiation, cigarettes per day, age at initiation of regular smoking, and smoking cessation. Genetic associations with CAD in patients with diabetes were extracted from another GWAS, which included 15,666 participants (3,968 CAD cases and 11,696 controls). The analyses were performed using the univariable and multivariable Mendelian randomization (MR) method.

**Results:**

MR analysis revealed that smoking initiation was positively related to CAD risk in patients with diabetes (OR = 1.322, 95% CI = 1.114 – 1.568, *P* = 0.001), but this association was attenuated when adjusted for cardiovascular risk factors (OR = 1.212, 95% CI = 1.008 – 1.457, *P* = 0.041). Age at initiation of regular smoking was negatively related to CAD in patients with diabetes (OR = 0.214, 95% CI = 0.070 – 0.656, *P* = 0.007), but this association became insignificant when adjusted for cardiovascular risk factors.

**Conclusions:**

This study supported the effect of smoking initiation on the risk of CAD in patients with diabetes.

## Introduction

Accompanied by increasing in obesity, aging and diabetes, the incidence and mortality of coronary artery disease (CAD) are increasing annually, which has become a public healthcare burden on a global scale (1). It is established that patients with diabetes are at remarkably higher risk of cardiovascular events than those without diabetes (2, 3). Therefore, the health systems community has paid great attention to the CAD prevention and treatment, especially in patients with diabetes (4, 5). It is crucial to reveal the causal risk factors and the underlying biological mechanisms for preventing CAD in patients with diabetes.

It is widely acknowledged that smoking is one of the major risk factors for angina pectoris, myocardial infarction and sudden death (6–8). A large scale-cohort study focused on a population with type 2 diabetes supported that smoking was the strongest predictor of death, but no evidence of significant relationship between smoking and CAD was found (2). However, there is a lack of strong evidence to clarify the causal relationship between smoking and CAD risk in patients with diabetes.

Mendelian randomization (MR) is an approach that employed to assess the causality between exposures and outcomes (9). Since genotype precedes phenotype, and alleles are randomly assigned at meiosis, this method is less susceptible to measurement error, confounding factors and reverse causality compared with traditional observational studies (10). Multivariable MR analyses and mediation analyses can help to explore the further mechanism underlying the relationships observed in univariable analyses (11, 12). In present study, we investigated the relationship between smoking and CAD risk in patients with diabetes using MR approach.

## Methods

### Study design

**Figure 1:**
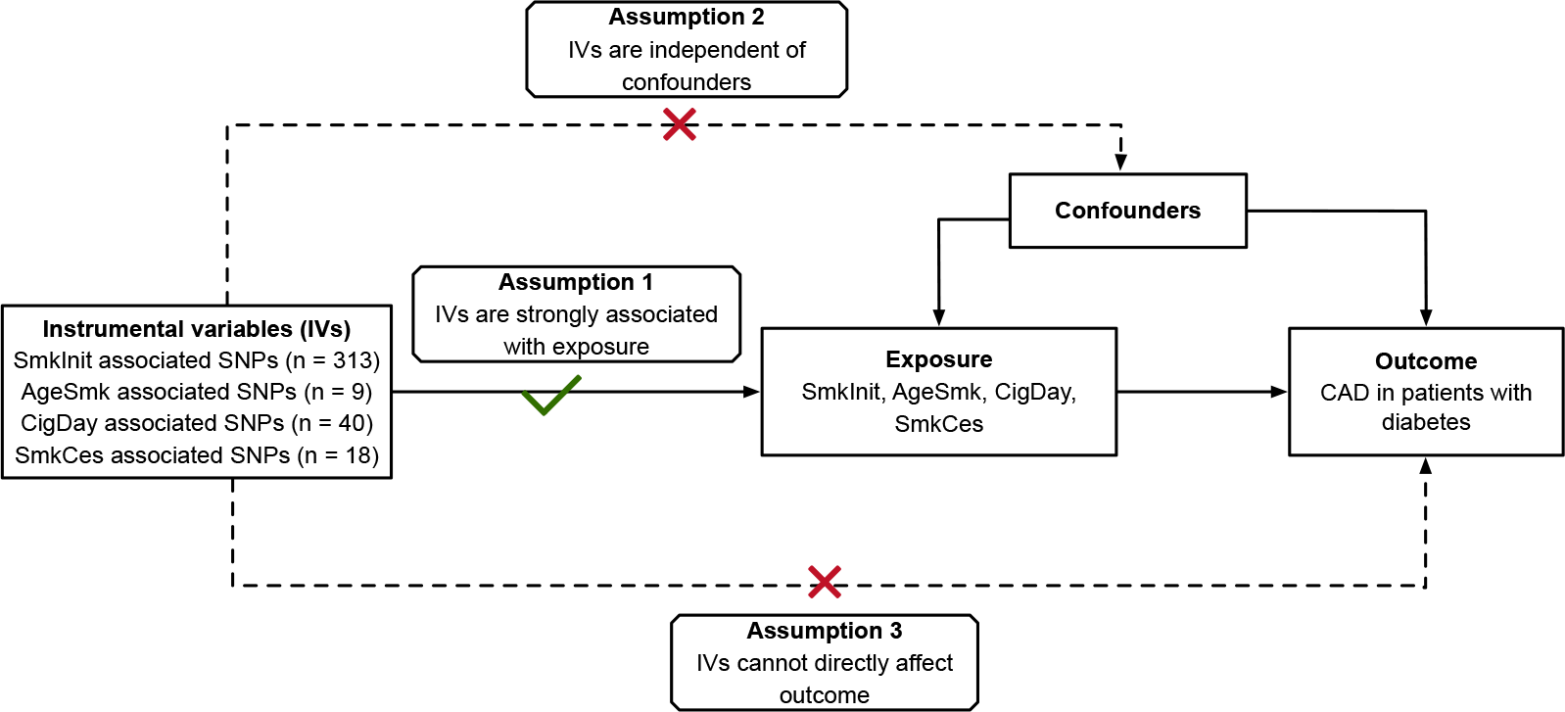
Conceptual schematics for the design of Mendelian randomization study. IV indicates instrumental variables; SmkInit, smoking initiation; AgeSmk, age at initiation of regular smoking; CigDay, cigarettes per day; SmkCes, smoking cessation; CAD, coronary artery disease.

We utilized a two-sample MR approach to evaluate the causal effect of genetic predicted smoking traits on the CAD risk in patients with diabetes. To do this, we selected single nucleotide polymorphisms (SNPs) as genetic instruments (IVs) for smoking traits, which must obey the following rules: (1) IVs must be robustly related to smoking traits; (2) IVs should not be associated with potential confounding factors; (3) IVs must influence the CAD risk in patients with diabetes only *via* the smoking traits (**Figure 1**).

### Data sources

Genetic signatures for smoking traits were obtained from a large genome-wide association study (GWAS), comprising of up to 1.2 million European-descent participants (13). Four smoking phenotypes were included in that study: smoking initiation, cigarettes per day, age at initiation of regular smoking, and smoking cessation. Summary-level data for genetic associations with CAD risk in patients with diabetes were obtained from a another GWAS, which included 15,666 European-descent participants with diabetes in UK biobank (3,968 CAD cases and 11,696 controls) (14). In addition, genetic associations with cardiovascular risk factors (e.g. body composition, blood pressure, serum lipids, as well as others) were also obtained from corresponding consortium or GWAS. Information on data sources was provided in **Table 1**. All studies included in the GWASs had been approved by an ethical review committee, and all the participants had provided informed consent. No additional ethics approval was required for current study.

**Table 1:** Overview of data sources.

Traits	No. of cases	No. of controls	Population	Consortium
Exposure				
SmkInit	NA	1,232,091	European	GSCAN (13)
AgeSmk	NA	341,427	European	GSCAN (13)
CigDay	NA	337,334	European	GSCAN (13)
SmkCes	NA	547,219	European	GSCAN (13)
Outcome				
CAD in diabetes	3,968	11,696	European	UK biobank (14)
Body composition				
BMI	NA	694,649	European	GIANT (15)
WHR	NA	694,649	European	GIANT (15)
Blood pressure				
SBP	NA	757,601	European	UK biobank and ICBP (16)
DBP	NA	757,601	European	UK biobank and ICBP (16)
PP	NA	757,601	European	UK biobank and ICBP (16)
Serum lipids				
HDL-C	NA	187,167	Mixed	UK biobank and GLGC (17)
LDL-C	NA	173,083	Mixed	UK biobank and GLGC (17)
TC	NA	187,365	Mixed	UK biobank and GLGC (17)
TG	NA	177,861	Mixed	UK biobank and GLGC (17)
Other related traits				
EA	NA	1,131,881	European	SSGAC (18)
Activity	NA	91,105	European	UK biobank (19)

SmkInit indicates smoking initiation; AgeSmk, age at initiation of regular smoking; CigDay, cigarettes per day; SmkCes, smoking cessation; CAD, coronary artery disease; BMI, body mass index; WHR, waist-to-hip ratio; SBP, systolic blood pressure; DBP, diastolic blood pressure; PP, pulse pressure; HDL-C, high-density lipoprotein cholesterol; LDL-C, low-density lipoprotein cholesterol; TC, total cholesterol; TG, triglyceride; EA, educational attainment.

### SNPs selection

We selected SNPs associated with smoking traits from the GWAS of smoking at a level of genome-wide significance (p < 5 × 10^−8^). The specific SNP was excluded when it was unavailable in the summary data for CAD risk in patients with diabetes. The selected SNPs were clumped (clumping window = 10Mb, clumping r2 cutoff = 0.01) using the PLINK clumping method. In total, 313 SNPs were used as IVs for smoking initiation, 9 SNPs for age at initiation of regular smoking, 40 SNPs for cigarettes per day, and 18 SNPs for smoking cessation. All the SNPs were valid (F-statistic > 10). **Table S1**–**S4** presented the detailed information for identified SNPs.

### Statistical analysis

We utilized the inverse variance weighted (IVW) method as our primary MR analysis. Specifically, the Wald estimator was applied to calculate the effect of each SNP, and the Delta method was applied to calculate the corresponding standard error (SE). Subsequently, we calculated the overall estimate following an IVW formula (20). In sensitivity analysis, we used the weighted median method, MR-Egger method and MR Pleiotropy Residual Sum and Outlier (MR-PRESSO) method as a complement. These methods could provide more robust results or correct for pleiotropy (21–23). In addition, Q-statistic was applied to assess the heterogeneity among SNPs, and the intercept of MR-Egger regression was used to appraise the potential directional pleiotropy (21, 22). *P* < 0.05 suggested existence of heterogeneity and pleiotropy, respectively. Moreover, funnel plots were generated to provide a visual inspection, in which symmetric graphics indicated absence of pleiotropy (24).

Multivariable MR analyses (25) were conducted to estimate the effect of smoking traits on CAD risk in patients with diabetes conditional on other cardiovascular risk factors. The adjusted factors in multivariable MR analyses included body mass index (BMI), waist-to-hip ratio (WHR), diastolic blood pressure (DBP), systolic blood pressure (SBP), pulse pressure (PP), low-density lipoprotein cholesterol (LDL-C), high-density lipoprotein cholesterol (HDL-C), total cholesterol (TC), triglyceride (TG), educational attainment (EA), and physical activity. Both the SNPs proxy for smoking trait and SNPs proxy for adjusted cardiovascular risk factor were used in multivariable MR, and the specific SNP was excluded when it was not available in the summary data. Also, these SNPs were clumped as described above. All analyses were performed using the R packages “TwoSampleMR” and “MendelianRandomization” in RStudio (R version 3.6.2). The statistical power was calculated on mRnd (**Table S5**) (26).

## Results

### The association of smoking traits with CAD risk in patients with diabetes

**Figure 2:**
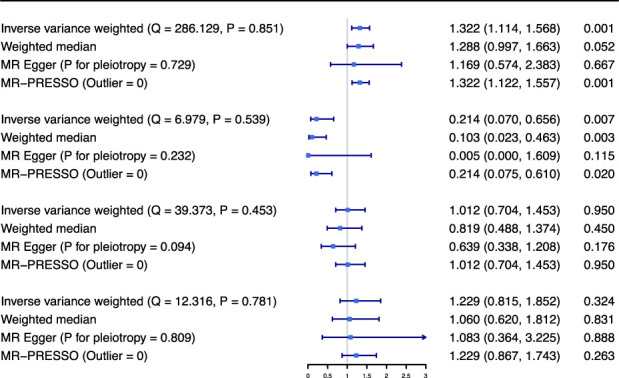
Mendelian randomization results for association between smoking traits and coronary artery disease in patients with diabetes. Odds ratio is scaled to per standard deviation increasement in genetically determined smoking initiation (about 10-12% increasement in the probability of being a regular smoker), age at initiation of regular smoking (about additional 0.31-0.50 years), cigarettes per day (about additional 2-3 cigarettes daily) and smoking cessation (about 3-5% increasement in the probability of being a current smoker). OR indicates odds ratio; CI, confidence interval; SmkInit, smoking initiation; AgeSmk, age at initiation of regular smoking; CigDay, cigarettes per day; SmkCes, smoking cessation; MR, Mendelian randomization; MR-PRESSO, MR Pleiotropy Residual Sum and Outlier.

**Figure 2** showed the MR results for the association of smoking traits with the CAD risk in patients with diabetes. The IVW analyses revealed that smoking initiation was positively related to the risk of CAD among patients with diabetes (odds ratio (OR) = 1.322, 95% confidence interval (CI) = 1.114 – 1.568, *P* = 0.001), while age at initiation of regular smoking was negatively related to the risk of CAD among patients with diabetes (OR = 0.214, 95% CI = 0.070 – 0.656, *P* = 0.007). However, no significant association was observed for smoking frequency (cigarette per day) and smoking cessation. The results for these associations in sensitivity analyses were similar to those of primary analysis. Q-statistic and the intercept of MR-Egger regression suggested no evidence of heterogeneity and horizonal pleiotropy, respectively. No outlier was detected in MR-PRESSO analysis. Symmetrical graphics in funnel plots presented in **Figure S1** provided another evidence against pleiotropy.

### The association of smoking initiation and age at initiation of regular smoking with cardiovascular risk factors

We next assessed if smoking initiation and age at initiation of regular smoking modulated other cardiovascular risk factors. The IVW analyses demonstrated that smoking initiation was positively associated with BMI, WHR, TG, while negatively associated with HDL-C and EA. In addition, it was found that age at initiation of regular smoking was negatively related to BMI, WHR, but positively associated with HDL-C and EA (**Table 2**).

**Table 2:** Association of smoking initiation and age at initiation of regular smoking with cardiovascular risk factors.

Traits	SmkInit			AgeSmk		
	SNPs	Beta (95% CI)	P	SNPs	Beta (95% CI)	P
BMI	313	0.178 (0.134, 0.221)	7.44E-16	9	-0.345 (-0.632, -0.058)	0.019
WHR	313	0.175 (0.146, 0.203)	3.29E-33	9	-0.278 (-0.527, -0.029)	0.029
SBP	309	-0.277 (-0.792, 0.238)	0.292	9	-0.869 (-5.868, 4.129)	0.733
DBP	310	-0.052 (-0.343,0.240)	0.728	9	-0.125 (-3.320, 3.070)	0.939
PP	309	-0.229 (-0.554, 0.097)	0.168	9	-0.756 (-2.803, 1.291)	0.469
HDL-C	150	-0.064 (-0.118, -0.010)	0.020	6	0.318 (0.072, 0.564)	0.011
LDL-C	150	0.006 (-0.042, 0.054)	0.815	6	-0.206 (-0.472, 0.060)	0.129
TC	150	0.019 (-0.032, 0.071)	0.460	6	-0.038 (-0.298, 0.222)	0.774
TG	150	0.093 (0.043, 0.142)	2.75E-04	6	-0.029 (-0.268, 0.210)	0.812
EA	311	-0.198 (-0.227, -0.169)	1.94E-41	9	0.491 (0.309, 0.673)	1.20E-07
Activity	313	-0.009 (-0.050, 0.032)	0.675	9	0.080 (-0.146, 0.307)	0.487

SmkInit indicates smoking initiation; AgeSmk, age at initiation of regular smoking; SNP, single-nucleotide polymorphism; CI, confidence interval; BMI, body mass index; WHR, waist-to-hip ratio; SBP, systolic blood pressure; DBP, diastolic blood pressure; PP, pulse pressure; HDL-C, high-density lipoprotein cholesterol; LDL-C, low-density lipoprotein cholesterol; TC, total cholesterol; TG, triglyceride; EA, educational attainment.

### The association of smoking initiation and age at initiation of regular smoking with CAD risk in patients with diabetes conditional on cardiovascular risk factors

In addition, we estimated the causal effect of smoking initiation and age at initiation of regular smoking on the risk of CAD in patients with diabetes, conditional on cardiovascular risk factors, using multivariable MR analyses.

**Figure 3:**
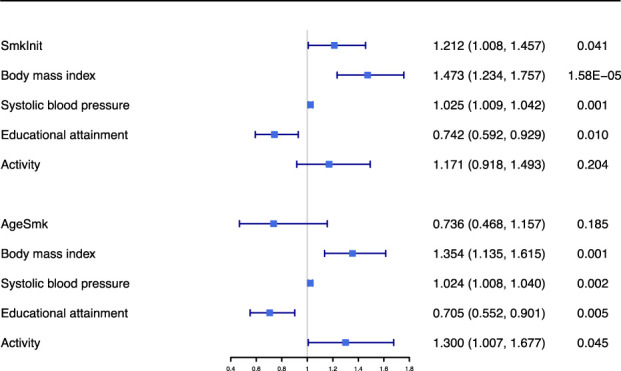
Multivariable Mendelian randomization results for association of smoking initiation and age at initiation of regular smoking with coronary artery disease in patients with diabetes when adjusted for body mass index, systolic blood pressure, educational attainment and activity. MVMR, multivariable Mendelian randomization; SmkInit, smoking initiation; AgeSmk, age at initiation of regular smoking.

After adjusting cardiovascular risk factor, robust association of smoking initiation with the risk of CAD in patients with diabetes was observed (**Table 3**). However, the effect of smoking initiation on the risk of CAD in patients with diabetes was attenuated when adjusted for BMI (significant) or EA (marginally significant). The association of smoking initiation with the risk of CAD in patients with diabetes was insignificant when adjusted for HDL-C or TG, which may be biased since around half of the selected SNPs proxy for smoking initiation were lost in this analysis. In the fully adjusted model, the effect of smoking initiation on the risk of CAD in patients with diabetes was attenuated but still significant (**Figure 3**).

**Table 3:** Multivariable Mendelian randomization results for association of smoking initiation and age at initiation of regular smoking with coronary artery disease in patients with diabetes.

Adjusted Trait	SmkInit-CAD in diabetes			AgeSmk-CAD in diabetes		
	SNPs	OR (95%CI)	P	SNPs	OR (95%CI)	P
BMI	813	1.220 (1.027, 1.450)	0.024	614	1.027 (0.570, 1.853)	0.928
WHR	558	1.281 (1.070, 1.535)	0.007	300	0.982 (0.467, 2.064)	0.962
SBP	502	1.354 (1.139, 1.609)	0.001	234	0.347 (0.140, 0.865)	0.023
DBP	547	1.328 (1.120, 1.575)	0.001	282	0.406 (0.197, 0.837)	0.015
PP	500	1.330 (1.121, 1.577)	0.001	225	0.219 (0.095, 0.502)	3.37E-04
HDL-C	201	1.244 (0.968, 1.598)	0.088	61	0.162 (0.056, 0.465)	0.001
LDL-C	178	1.309 (1.018, 1.682)	0.036	35	0.148 (0.032, 0.690)	0.015
TC	186	1.300 (1.011, 1.670)	0.040	44	0.142 (0.043, 0.468)	0.001
TG	175	1.247 (0.969, 1.606)	0.085	31	0.191 (0.052, 0.705)	0.013
EA	987	1.201 (0.999, 1.444)	0.052	820	0.592 (0.337, 1.038)	0.067
Activity	314	1.322 (1.115, 1.568)	0.001	10	0.190 (0.061, 0.587)	0.004
ALL traits (BMI^a^, SBP^b^, EA, Activity) ^c^	1444	1.212 (1.008, 1.457)	0.041	1328	0.736 (0.468, 1.157)	0.185

SmkInit indicates smoking initiation; CAD, coronary artery disease; AgeSmk, age at initiation of regular smoking; SNP, single-nucleotide polymorphism; OR, odds ratio; CI, confidence interval; BMI, body mass index; WHR, waist-to-hip ratio; SBP, systolic blood pressure; DBP, diastolic blood pressure; PP, pulse pressure; HDL-C, high-density lipoprotein cholesterol; LDL-C, low-density lipoprotein cholesterol; TC, total cholesterol; TG, triglyceride; EA, educational attainment.

^a^ Restricted to BMI to avoid collinearity with WHR.

^b^ Restricted to SBP to avoid collinearity with DBP and PP.

^c^ Lipid trait was not included in this adjusted model, since around half of the selected SNPs were not available in the lipid summary datasets.

The relationship between age at initiation of regular smoking and susceptibility to CAD in patients with diabetes was significant when adjusted for blood pressure trait, lipid trait or activity, but insignificant when adjusted for body composition trait or EA (**Table 3**). This result indicated that the effect of age at initiation of regular smoking on the risk of CAD in patients with diabetes might be explained by body composition trait and EA. Besides, the association of age at initiation of regular smoking with the risk of CAD in patients with diabetes was not significant in the fully adjusted model (**Table 3** and **Figure 3**).

## Discussion

This is a two-sample MR study to appraise the causal relationship between smoking traits and the risk of CAD among patients with diabetes. Our study supported that genetically determined smoking initiation was positively related to the risk of CAD among patients with diabetes, and that age at initiation of regular smoking was negatively related to the risk of CAD among patients with diabetes. However, smoking frequency (cigarette per day) and smoking cessation were not associated with CAD risk in patients with diabetes. Using multivariable MR analyses, our study suggested that smoking initiation was independently associated with CAD in patients with diabetes, whereas the effect was attenuated when adjusted for BMI or EA. Besides, the effect of age at initiation of regular smoking may be explained by other cardiovascular risk factors.

Smoking is a modifiable risk factor for many chronic diseases, e.g. cancer, chronic obstructive lung disease, asthma, CAD and diabetes (6, 27–30). The adverse effects of smoking on CAD have been generally under recognized, and more and more research has been devoted to studying the potential mechanisms of the relationship. Nicotine can result in harm to the cardiovascular system by increasing the level of free radicals and other toxic substances, which may be related to an increased risk of CAD (31). Cigarette smoking could cause complex pathophysiological process within the blood vessel wall, which may work by influencing the following four pathways (32): (a) lipid oxidation; (b) stimulation of vascular smooth muscle cell proliferation; (c) promotion of the expression of inflammatory factors; and (d) weakened endothelium-mediated platelet inhibition with a tendency for thrombosis. Furthermore, cigarette smoking may increase vasoconstriction and myocardial contractility, resulting in increased myocardial work, myocardial oxygen consumption and a reduction in coronary blood flow as well, which will provoke acute cardiovascular events (33).

Randomized controlled trials (RCTs) are determined as the gold standard in clinical research to reveal the causality (34). However, RCTs remain impractical, because of extraordinary cost of time and money (35). Since genotype precedes phenotype, and alleles are randomly assigned at meiosis, MR approach evades reverse causality and is less susceptible to confoundings, which may serve as a timely alternative approach compared with conventional observational studies (25, 36). Several MR studies have been conducted previously to investigate the association between smoking and diabetes or cardiovascular disease. Yuan et al. found a strong association between smoking and diabetes (37). A large‐scale MR study supported the cause-effect relationship between smoking and multiple cardiovascular and cerebrovascular diseases among the general population, in particular, CAD, transient ischaemic attack, ischaemic stroke, heart failure, peripheral arterial disease, abdominal aortic aneurysm, and arterial hypertension (38). Levin et al. in their work found that genetic liability to smoking was a strong risk factor for atherosclerotic cardiovascular diseases, including CAD, peripheral artery disease, and large-artery stroke (39). Another study on general populations using MR approach has demonstrated that current smokers and smoking initiation are causes of CAD and myocardial infarction (40). But people who smoke more cigarettes per day have no causal association with CAD (40). Thom et al. used the MR approach to determine whether BMI mediate the effect of smoking on type 2 diabetes and CAD, respectively (41). Their results supported that smoking initiation increased a risk of type 2 diabetes and CAD, and BMI mediated the effect of smoking on type 2 diabetes but not CAD (41).

In this study, we focused on the population with diabetes. Consistent with findings from previous studies among the general population (40, 41), our findings supported that smoking initiation was positively related to CAD risk in patients with diabetes. Besides, we found that age at initiation of regular smoking was negatively associated with CAD risk. Unlike previous studies (40), we found that the effect of smoking initiation on CAD risk were partially explained by BMI or EA, as we focused on patients with diabetes. Likewise, the effect of age at initiation of regular smoking might be explained by body composition trait or EA. Further investigation was warranted to identify how smoking risk portends an increased risk of CAD among patients with diabetes.

The highlight of present study lies in the design of MR analysis, which used smoking-related SNPs and SNPs-CAD summary data from large-scale GWASs. This could reduce confounding and reverse causation compared with conventional observational studies. Large sample size is another strength, which allows us to examine the hypothesis more precisely. In addition, the study participants are restricted to European-descent, which minimizes the population stratification bias. Furthermore, multivariable MR analyses were used to avoid the bias of confounding and reverse causation, which could be helpful in explaining relationships observed in univariable analyses.

There are several limitations that warrant discussion. First, it was difficult to entirely rule out the potential pleiotropy, which was an inherent limitation to the MR analysis. However, we did adjust for other traits in our multivariate analyses and the sensitivity analyses yielded robust results. Second, as seen in the work by Xue et al., behavior traits are subject to bias by misreports and longitudinal changes in GWAS and follow-up analyses (42). We should be cautious about interpretation of the presented causal associations, since smoking traits are behavior characteristics. Third, when we analyzed the effect of age at initiation of regular smoking, cigarettes per day, smoking cessation on the risk of CAD in patients with diabetes, the statistical power did not reach 80% (**Table S5**). It may be caused by the low variance in exposures explained by the selected SNPs and the insufficient sample size. Forth, there is some degree of overlap between the participants included in the GWAS for smoking traits and coronary artery disease in patients with diabetes, which may cause bias. Fifth, around half of the selected SNPs were not available in the summary data of lipid trait, thus we exclude this trait in the fully adjusted model, which can also cause bias. Sixth, the current study only investigated the association of smoking behaviors with CAD risk among patients with diabetes from a genetic viewpoint. Finally, since the study participants were of European ancestry, whether these findings could be generalizable to other populations remained unclear.

## Conclusions

Our findings provided evidence to support the effect of smoking initiation on the risk of CAD in patients with diabetes.

## Data availability statement

The original contributions presented in the study are included in the article/**Supplementary Material**. Further inquiries can be directed to the corresponding author.

## Author contributions

SZC, WBZ and GSF conceived the study, participated in the design. SZC, FKY, TX, YW and KJZ performed the statistical analyses, and drafted the manuscript. SZC, WBZ and GSF revised the paper. All authors contributed to the article and approved the submitted version.
