# Supplementary material for: Smoking and coronary artery disease risk in patients with diabetes: A Mendelian randomization study
Source: Front Immunol. 2023 Jan 26;14:891947. doi: 10.3389/fimmu.2023.891947 (PMC9910331; doi:10.3389/fimmu.2023.891947)
Supplement: Supplementary file 1 [file DataSheet_1.docx]

**Supplement Materials**

**Table S1.** Characteristics of SNPs and their associations with SmkInit and CAD in patients with diabetes.

**Table S2.** Characteristics of SNPs and their associations with AgeSmk and CAD in patients with diabetes.

**Table S3.** Characteristics of SNPs and their associations with CigDay and CAD in patients with diabetes.

**Table S4.** Characteristics of SNPs and their associations with SmkCes and CAD in patients with diabetes.

**Table S5.** Power calculation for the Mendelian randomization analyses.

**Figure S1.** Funnel plots of the Mendelian randomization estimate for the associations between smoking traits and coronary artery disease in patients with diabetes.

**Table S1.** Characteristics of SNPs and their associations with SmkInit and CAD in patients with diabetes.

| SNP | Chr | Pos | EA | OA | EAF | F | SmkInit | | | CAD in diabetes | | |
| --- | --- | --- | --- | --- | --- | --- | --- | --- | --- | --- | --- | --- |
|  |  |  |  |  |  |  | Beta | SE | P | Beta | SE | P |
| rs12130857 | 1 | 7791461 | A | G | 0.325 | 175 | -0.0180 | 0.0027 | 3.65E-11 | -0.0240 | 0.0285 | 0.4009 |
| rs301807 | 1 | 8484823 | G | A | 0.570 | 196 | 0.0180 | 0.0026 | 2.5E-12 | 0.1072 | 0.0271 | 0.0001 |
| rs3820277 | 1 | 18436657 | T | G | 0.526 | 218 | -0.0188 | 0.0026 | 1.57E-13 | 0.0149 | 0.0268 | 0.5784 |
| rs1889571 | 1 | 32195819 | G | T | 0.131 | 138 | 0.0222 | 0.0038 | 4.19E-09 | -0.0485 | 0.0400 | 0.2258 |
| rs10914684 | 1 | 33795572 | A | G | 0.324 | 135 | -0.0158 | 0.0027 | 6.32E-09 | 0.0269 | 0.0284 | 0.3440 |
| rs2637869 | 1 | 38757237 | A | G | 0.297 | 171 | 0.0182 | 0.0028 | 6.54E-11 | -0.0363 | 0.0293 | 0.2159 |
| rs12755632 | 1 | 41776623 | G | A | 0.316 | 126 | -0.0154 | 0.0027 | 1.93E-08 | 0.0145 | 0.0284 | 0.6113 |
| rs951740 | 1 | 44011737 | A | G | 0.625 | 504 | 0.0295 | 0.0026 | 3.82E-29 | 0.0104 | 0.0278 | 0.7074 |
| rs925524 | 1 | 46496709 | G | A | 0.710 | 123 | 0.0156 | 0.0028 | 2.94E-08 | -0.0504 | 0.0295 | 0.0870 |
| rs12022778 | 1 | 50603995 | C | A | 0.202 | 286 | 0.0268 | 0.0032 | 3.18E-17 | 0.0055 | 0.0326 | 0.8648 |
| rs4912332 | 1 | 58815243 | T | C | 0.491 | 123 | 0.0141 | 0.0025 | 2.94E-08 | 0.0548 | 0.0267 | 0.0402 |
| rs1937443 | 1 | 66469643 | G | C | 0.563 | 253 | 0.0204 | 0.0026 | 1.79E-15 | 0.0412 | 0.0270 | 0.1266 |
| rs12740789 | 1 | 72752073 | A | G | 0.178 | 293 | -0.0285 | 0.0033 | 1.18E-17 | -0.0315 | 0.0345 | 0.3611 |
| rs10789369 | 1 | 73824909 | G | A | 0.615 | 321 | -0.0234 | 0.0026 | 3.39E-19 | 0.0062 | 0.0276 | 0.8209 |
| rs1514176 | 1 | 74991596 | A | G | 0.580 | 224 | -0.0193 | 0.0026 | 7.67E-14 | 0.0110 | 0.0270 | 0.6847 |
| rs10873871 | 1 | 76689019 | G | A | 0.207 | 123 | 0.0175 | 0.0031 | 2.82E-08 | 0.0504 | 0.0322 | 0.1169 |
| rs11162019 | 1 | 87913176 | T | C | 0.363 | 137 | -0.0155 | 0.0026 | 5.06E-09 | 0.0108 | 0.0278 | 0.6987 |
| rs1008078 | 1 | 91189731 | T | C | 0.402 | 308 | 0.0228 | 0.0026 | 1.63E-18 | 0.0045 | 0.0272 | 0.8679 |
| rs12027999 | 1 | 154206358 | C | T | 0.120 | 154 | -0.0244 | 0.0039 | 5.33E-10 | -0.0255 | 0.0397 | 0.5199 |
| rs45444697 | 1 | 155034632 | G | C | 0.212 | 160 | 0.0197 | 0.0031 | 2.72E-10 | -0.0493 | 0.0319 | 0.1221 |
| rs2901785 | 1 | 174104743 | A | G | 0.446 | 182 | -0.0173 | 0.0026 | 1.47E-11 | -0.0226 | 0.0268 | 0.3986 |
| rs147052174 | 1 | 179783167 | T | G | 0.017 | 161 | 0.0623 | 0.0098 | 2.3E-10 | 0.0118 | 0.0956 | 0.9018 |
| rs35656245 | 1 | 190957480 | A | G | 0.276 | 125 | 0.0159 | 0.0029 | 2.23E-08 | 0.0007 | 0.0302 | 0.9811 |
| rs12739243 | 1 | 210302043 | C | T | 0.221 | 192 | -0.0213 | 0.0031 | 4.45E-12 | -0.0020 | 0.0327 | 0.9518 |
| rs12563365 | 1 | 236872829 | A | G | 0.556 | 167 | 0.0166 | 0.0026 | 1.05E-10 | -0.0081 | 0.0269 | 0.7631 |
| rs876793 | 1 | 237852083 | C | T | 0.349 | 180 | -0.0179 | 0.0027 | 5.69E-11 | 0.0007 | 0.0280 | 0.9805 |
| rs114976176 | 2 | 264621 | C | A | 0.352 | 135 | -0.0155 | 0.0027 | 6.04E-09 | 0.0218 | 0.0278 | 0.4328 |
| rs62106258 | 2 | 417167 | C | T | 0.047 | 230 | -0.0455 | 0.0060 | 3.33E-14 | -0.0787 | 0.0662 | 0.2340 |
| rs6731872 | 2 | 624205 | G | T | 0.826 | 354 | 0.0316 | 0.0034 | 5.35E-21 | -0.0308 | 0.0359 | 0.3917 |
| rs1022376 | 2 | 22067213 | C | T | 0.516 | 134 | -0.0147 | 0.0026 | 1.66E-08 | 0.0084 | 0.0268 | 0.7531 |
| rs61533748 | 2 | 22582968 | C | T | 0.384 | 177 | 0.0174 | 0.0026 | 2.82E-11 | -0.0152 | 0.0274 | 0.5781 |
| rs72790288 | 2 | 29513404 | A | G | 0.028 | 140 | -0.0455 | 0.0077 | 3.28E-09 | 0.2035 | 0.0751 | 0.0067 |
| rs2710634 | 2 | 32808804 | C | T | 0.521 | 194 | -0.0178 | 0.0026 | 3.36E-12 | 0.0005 | 0.0268 | 0.9858 |
| rs62137126 | 2 | 44250149 | G | A | 0.121 | 147 | -0.0237 | 0.0039 | 1.31E-09 | -0.0384 | 0.0409 | 0.3482 |
| rs1004787 | 2 | 45159091 | A | G | 0.552 | 492 | 0.0284 | 0.0026 | 1.11E-28 | 0.0506 | 0.0268 | 0.0593 |
| rs7598402 | 2 | 50735943 | G | C | 0.492 | 134 | -0.0147 | 0.0025 | 7.38E-09 | -0.0338 | 0.0269 | 0.2087 |
| rs10490159 | 2 | 51341259 | T | C | 0.394 | 175 | 0.0172 | 0.0026 | 3.86E-11 | 0.0279 | 0.0273 | 0.3065 |
| rs1518393 | 2 | 58171220 | C | A | 0.619 | 165 | 0.0169 | 0.0026 | 1.3E-10 | 0.0256 | 0.0276 | 0.3536 |
| rs17616642 | 2 | 59022210 | G | A | 0.247 | 126 | -0.0166 | 0.0030 | 2.1E-08 | -0.0015 | 0.0307 | 0.9606 |
| rs2539706 | 2 | 59819545 | A | G | 0.530 | 162 | 0.0162 | 0.0026 | 1.95E-10 | 0.0124 | 0.0269 | 0.6459 |
| rs1863161 | 2 | 60139524 | A | G | 0.561 | 143 | 0.0153 | 0.0026 | 2.34E-09 | -0.0186 | 0.0268 | 0.4872 |
| rs359247 | 2 | 60477052 | T | A | 0.639 | 276 | 0.0220 | 0.0027 | 9.89E-17 | 0.0083 | 0.0280 | 0.7662 |
| rs62180324 | 2 | 63416606 | A | G | 0.212 | 157 | -0.0195 | 0.0031 | 3.91E-10 | -0.0042 | 0.0323 | 0.8959 |
| rs6750107 | 2 | 80748807 | A | G | 0.387 | 124 | 0.0146 | 0.0026 | 2.6E-08 | 0.0230 | 0.0274 | 0.4019 |
| rs12714017 | 2 | 80999398 | C | T | 0.511 | 146 | 0.0154 | 0.0026 | 3.65E-09 | -0.0618 | 0.0268 | 0.0209 |
| rs56208390 | 2 | 83247997 | G | A | 0.123 | 124 | 0.0216 | 0.0039 | 2.68E-08 | -0.0913 | 0.0423 | 0.0310 |
| rs11692435 | 2 | 98275354 | A | G | 0.085 | 120 | 0.0251 | 0.0046 | 4.47E-08 | -0.0069 | 0.0497 | 0.8902 |
| rs13392222 | 2 | 100672408 | C | A | 0.139 | 162 | -0.0234 | 0.0037 | 1.93E-10 | 0.0816 | 0.0375 | 0.0296 |
| rs1901477 | 2 | 104126983 | G | A | 0.511 | 571 | 0.0304 | 0.0026 | 2.07E-31 | 0.0530 | 0.0265 | 0.0454 |
| rs3811038 | 2 | 113240183 | C | T | 0.279 | 182 | 0.0191 | 0.0028 | 1.58E-11 | -0.0232 | 0.0300 | 0.4402 |
| rs34399632 | 2 | 137571174 | G | A | 0.232 | 164 | 0.0194 | 0.0030 | 1.46E-10 | -0.0206 | 0.0304 | 0.4970 |
| rs6756212 | 2 | 146140132 | T | C | 0.535 | 704 | -0.0339 | 0.0026 | 3.49E-40 | 0.0179 | 0.0270 | 0.5069 |
| rs16826827 | 2 | 147825689 | C | T | 0.124 | 132 | -0.0222 | 0.0039 | 9.17E-09 | -0.0295 | 0.0402 | 0.4629 |
| rs1445649 | 2 | 155682556 | C | T | 0.538 | 259 | 0.0206 | 0.0026 | 8.48E-16 | 0.0314 | 0.0269 | 0.2429 |
| rs12474587 | 2 | 162802993 | T | G | 0.429 | 355 | 0.0242 | 0.0026 | 4.83E-21 | -0.0212 | 0.0271 | 0.4335 |
| rs357304 | 2 | 164862639 | C | T | 0.727 | 136 | 0.0167 | 0.0029 | 5.4E-09 | -0.0319 | 0.0302 | 0.2910 |
| rs13007361 | 2 | 166250244 | A | G | 0.208 | 125 | 0.0175 | 0.0031 | 2.29E-08 | 0.0171 | 0.0329 | 0.6033 |
| rs7600835 | 2 | 172521827 | A | G | 0.342 | 127 | -0.0151 | 0.0027 | 1.8E-08 | 0.0031 | 0.0284 | 0.9119 |
| rs6750529 | 2 | 182027603 | T | C | 0.744 | 186 | 0.0199 | 0.0029 | 9.26E-12 | 0.0199 | 0.0304 | 0.5134 |
| rs17229285 | 2 | 199523122 | T | C | 0.505 | 148 | -0.0155 | 0.0025 | 1.27E-09 | -0.0326 | 0.0268 | 0.2238 |
| rs3115418 | 2 | 200936399 | C | T | 0.454 | 124 | -0.0142 | 0.0026 | 2.79E-08 | -0.0171 | 0.0269 | 0.5242 |
| rs62193862 | 2 | 202843875 | A | G | 0.100 | 126 | 0.0238 | 0.0042 | 1.99E-08 | -0.0291 | 0.0446 | 0.5144 |
| rs4674916 | 2 | 225365635 | A | C | 0.328 | 176 | -0.0180 | 0.0027 | 3.06E-11 | -0.0554 | 0.0289 | 0.0553 |
| rs4674993 | 2 | 226332033 | G | A | 0.200 | 227 | -0.0240 | 0.0032 | 4.85E-14 | 0.0115 | 0.0337 | 0.7333 |
| rs11713899 | 3 | 2365026 | C | A | 0.171 | 122 | 0.0187 | 0.0034 | 3.15E-08 | -0.0420 | 0.0357 | 0.2403 |
| rs748832 | 3 | 16851202 | G | A | 0.371 | 170 | 0.0172 | 0.0026 | 6.6E-11 | -0.0122 | 0.0276 | 0.6581 |
| rs10446419 | 3 | 25725501 | G | A | 0.207 | 155 | -0.0196 | 0.0031 | 5.05E-10 | -0.0328 | 0.0332 | 0.3234 |
| rs3172494 | 3 | 48731487 | T | G | 0.115 | 213 | -0.0291 | 0.0040 | 3.4E-13 | -0.0855 | 0.0448 | 0.0566 |
| rs2526390 | 3 | 50192760 | T | C | 0.334 | 230 | 0.0205 | 0.0027 | 3.62E-14 | 0.0201 | 0.0280 | 0.4715 |
| rs2276825 | 3 | 52886605 | C | T | 0.245 | 162 | 0.0189 | 0.0030 | 1.89E-10 | 0.0337 | 0.0309 | 0.2754 |
| rs2306866 | 3 | 53766212 | T | A | 0.614 | 162 | -0.0167 | 0.0026 | 1.89E-10 | -0.0164 | 0.0273 | 0.5471 |
| rs73831818 | 3 | 55988394 | G | A | 0.057 | 136 | 0.0320 | 0.0055 | 5.46E-09 | 0.1073 | 0.0539 | 0.0466 |
| rs7640107 | 3 | 59966156 | T | C | 0.431 | 122 | -0.0142 | 0.0026 | 3.46E-08 | 0.0399 | 0.0271 | 0.1409 |
| rs2734390 | 3 | 60459291 | G | A | 0.372 | 126 | 0.0148 | 0.0026 | 2.09E-08 | -0.0262 | 0.0280 | 0.3499 |
| rs221988 | 3 | 64234307 | C | A | 0.384 | 129 | -0.0149 | 0.0026 | 1.43E-08 | -0.0265 | 0.0278 | 0.3404 |
| rs11128203 | 3 | 71064431 | A | T | 0.530 | 256 | 0.0204 | 0.0026 | 1.29E-15 | 0.0009 | 0.0268 | 0.9736 |
| rs62246017 | 3 | 71483084 | A | G | 0.323 | 141 | -0.0162 | 0.0027 | 3.03E-09 | -0.0113 | 0.0291 | 0.6972 |
| rs4543050 | 3 | 74954560 | T | A | 0.816 | 182 | 0.0222 | 0.0033 | 1.45E-11 | -0.0257 | 0.0349 | 0.4623 |
| rs6782116 | 3 | 77176032 | T | C | 0.415 | 128 | -0.0147 | 0.0026 | 1.46E-08 | -0.0127 | 0.0272 | 0.6411 |
| rs13066050 | 3 | 81325861 | T | C | 0.208 | 144 | 0.0188 | 0.0031 | 1.93E-09 | -0.0433 | 0.0331 | 0.1910 |
| rs12633090 | 3 | 83241365 | C | G | 0.182 | 194 | -0.0230 | 0.0033 | 3.16E-12 | -0.0368 | 0.0350 | 0.2930 |
| rs1549979 | 3 | 85460131 | T | C | 0.615 | 351 | -0.0245 | 0.0026 | 8.8E-21 | -0.0325 | 0.0272 | 0.2321 |
| rs6437769 | 3 | 107997514 | T | C | 0.581 | 121 | 0.0142 | 0.0026 | 3.74E-08 | 0.0175 | 0.0272 | 0.5195 |
| rs9288999 | 3 | 114147927 | A | G | 0.735 | 146 | 0.0174 | 0.0029 | 1.5E-09 | -0.0188 | 0.0310 | 0.5453 |
| rs6438436 | 3 | 117822149 | T | C | 0.816 | 226 | 0.0247 | 0.0033 | 5.33E-14 | 0.0255 | 0.0353 | 0.4697 |
| rs9826984 | 3 | 131945722 | A | G | 0.542 | 121 | -0.0141 | 0.0026 | 3.87E-08 | 0.0136 | 0.0267 | 0.6114 |
| rs2279829 | 3 | 147106319 | T | C | 0.216 | 126 | -0.0174 | 0.0031 | 2.05E-08 | 0.0186 | 0.0324 | 0.5652 |
| rs2319545 | 3 | 147719648 | A | C | 0.149 | 169 | 0.0232 | 0.0036 | 8.3E-11 | 0.0083 | 0.0366 | 0.8204 |
| rs10935779 | 3 | 149543102 | T | C | 0.415 | 123 | -0.0143 | 0.0026 | 2.95E-08 | -0.0542 | 0.0268 | 0.0432 |
| rs1714521 | 3 | 158284861 | C | A | 0.411 | 158 | -0.0163 | 0.0026 | 3.07E-10 | 0.0229 | 0.0270 | 0.3960 |
| rs1449012 | 3 | 159048333 | T | C | 0.463 | 145 | -0.0154 | 0.0026 | 1.77E-09 | -0.0196 | 0.0267 | 0.4620 |
| rs9850597 | 3 | 161761866 | A | G | 0.816 | 128 | -0.0186 | 0.0033 | 1.65E-08 | -0.0021 | 0.0347 | 0.9511 |
| rs1187820 | 3 | 173072584 | T | C | 0.439 | 124 | -0.0143 | 0.0026 | 2.69E-08 | -0.0100 | 0.0272 | 0.7127 |
| rs16828799 | 3 | 173353739 | T | G | 0.156 | 127 | 0.0198 | 0.0035 | 1.83E-08 | -0.0047 | 0.0372 | 0.8999 |
| rs9841807 | 3 | 175718927 | T | C | 0.273 | 129 | 0.0163 | 0.0029 | 1.35E-08 | 0.0041 | 0.0301 | 0.8922 |
| rs7631379 | 3 | 181409057 | C | T | 0.206 | 174 | 0.0208 | 0.0032 | 3.94E-11 | 0.0013 | 0.0328 | 0.9689 |
| rs4140932 | 4 | 15458598 | A | T | 0.431 | 119 | -0.0140 | 0.0026 | 4.89E-08 | 0.0112 | 0.0271 | 0.6797 |
| rs59537158 | 4 | 28246049 | T | C | 0.214 | 210 | 0.0225 | 0.0031 | 4.62E-13 | 0.0177 | 0.0317 | 0.5766 |
| rs55944129 | 4 | 29082156 | C | T | 0.267 | 149 | -0.0176 | 0.0029 | 1.06E-09 | 0.0026 | 0.0304 | 0.9312 |
| rs58400863 | 4 | 31184484 | A | G | 0.347 | 227 | -0.0202 | 0.0027 | 4.89E-14 | -0.0108 | 0.0282 | 0.7028 |
| rs7657022 | 4 | 35501032 | G | A | 0.489 | 206 | 0.0183 | 0.0025 | 7.34E-13 | -0.0024 | 0.0268 | 0.9291 |
| rs112725451 | 4 | 68017710 | T | C | 0.169 | 236 | 0.0261 | 0.0034 | 1.65E-14 | 0.0479 | 0.0355 | 0.1775 |
| rs1160685 | 4 | 94052854 | G | C | 0.450 | 143 | 0.0153 | 0.0026 | 2.31E-09 | -0.0219 | 0.0270 | 0.4166 |
| rs1435479 | 4 | 94550450 | T | G | 0.287 | 136 | 0.0164 | 0.0028 | 5.68E-09 | 0.0117 | 0.0289 | 0.6847 |
| rs3934797 | 4 | 112467612 | A | G | 0.182 | 166 | -0.0213 | 0.0033 | 1.12E-10 | -0.0103 | 0.0337 | 0.7599 |
| rs71602617 | 4 | 136406155 | T | C | 0.216 | 132 | -0.0178 | 0.0032 | 2.1E-08 | 0.0143 | 0.0327 | 0.6609 |
| rs7696257 | 4 | 137474783 | A | G | 0.366 | 134 | 0.0153 | 0.0026 | 6.78E-09 | -0.0280 | 0.0282 | 0.3210 |
| rs13109980 | 4 | 140886963 | A | G | 0.326 | 266 | -0.0222 | 0.0027 | 3.37E-16 | 0.0210 | 0.0285 | 0.4604 |
| rs1116690 | 4 | 143510148 | G | A | 0.742 | 125 | 0.0163 | 0.0029 | 2.16E-08 | -0.0208 | 0.0304 | 0.4950 |
| rs13110073 | 4 | 147797913 | C | T | 0.395 | 358 | -0.0246 | 0.0026 | 3.24E-21 | -0.0310 | 0.0275 | 0.2591 |
| rs62340589 | 4 | 176875795 | C | G | 0.201 | 120 | 0.0174 | 0.0032 | 4.31E-08 | 0.0040 | 0.0331 | 0.9042 |
| rs12517438 | 5 | 30842054 | G | T | 0.538 | 144 | 0.0154 | 0.0026 | 1.89E-09 | 0.0458 | 0.0268 | 0.0878 |
| rs35375873 | 5 | 43190647 | C | G | 0.110 | 176 | -0.0270 | 0.0041 | 3.29E-11 | 0.0200 | 0.0428 | 0.6407 |
| rs986714 | 5 | 50821338 | T | A | 0.445 | 156 | -0.0160 | 0.0026 | 4.13E-10 | 0.0037 | 0.0270 | 0.8913 |
| rs71592686 | 5 | 60121271 | C | T | 0.274 | 211 | 0.0207 | 0.0029 | 3.85E-13 | -0.0231 | 0.0300 | 0.4407 |
| rs2028269 | 5 | 79308315 | A | G | 0.399 | 154 | 0.0162 | 0.0026 | 5.19E-10 | -0.0101 | 0.0273 | 0.7098 |
| rs6874731 | 5 | 80263865 | G | T | 0.484 | 144 | 0.0153 | 0.0025 | 1.83E-09 | -0.0105 | 0.0272 | 0.7006 |
| rs6452785 | 5 | 87685500 | T | C | 0.474 | 444 | -0.0269 | 0.0026 | 4.69E-26 | 0.0468 | 0.0270 | 0.0829 |
| rs10805858 | 5 | 88873832 | T | A | 0.335 | 180 | 0.0181 | 0.0027 | 1.88E-11 | -0.0056 | 0.0284 | 0.8448 |
| rs42417 | 5 | 94198290 | T | C | 0.691 | 151 | 0.0169 | 0.0028 | 8.27E-10 | -0.0220 | 0.0290 | 0.4472 |
| rs72780746 | 5 | 103929588 | C | T | 0.173 | 234 | -0.0258 | 0.0034 | 2.05E-14 | 0.0095 | 0.0358 | 0.7905 |
| rs10060196 | 5 | 106455988 | A | C | 0.581 | 201 | 0.0183 | 0.0026 | 1.29E-12 | 0.0066 | 0.0269 | 0.8056 |
| rs72789626 | 5 | 106825618 | A | T | 0.136 | 190 | -0.0256 | 0.0037 | 5.13E-12 | 0.0332 | 0.0387 | 0.3913 |
| rs17165769 | 5 | 107365642 | G | A | 0.395 | 150 | 0.0159 | 0.0026 | 9.56E-10 | 0.0076 | 0.0274 | 0.7822 |
| rs329124 | 5 | 133865452 | G | A | 0.428 | 162 | -0.0164 | 0.0026 | 1.96E-10 | -0.0326 | 0.0271 | 0.2277 |
| rs1385108 | 5 | 154839646 | T | C | 0.239 | 157 | 0.0187 | 0.0030 | 3.84E-10 | 0.0457 | 0.0310 | 0.1410 |
| rs1173461 | 5 | 157707571 | T | C | 0.327 | 150 | 0.0166 | 0.0027 | 9.51E-10 | -0.0116 | 0.0285 | 0.6846 |
| rs11956866 | 5 | 161018271 | G | T | 0.567 | 133 | -0.0148 | 0.0026 | 7.82E-09 | 0.0154 | 0.0269 | 0.5662 |
| rs3909281 | 5 | 165096435 | G | T | 0.536 | 272 | 0.0211 | 0.0026 | 1.62E-16 | -0.0153 | 0.0270 | 0.5702 |
| rs3843905 | 5 | 165427280 | T | C | 0.403 | 136 | -0.0151 | 0.0026 | 5.41E-09 | 0.0011 | 0.0274 | 0.9686 |
| rs6890961 | 5 | 166778503 | T | C | 0.624 | 216 | -0.0193 | 0.0026 | 2.13E-13 | 0.0208 | 0.0278 | 0.4550 |
| rs4044321 | 5 | 166989513 | G | A | 0.644 | 290 | -0.0226 | 0.0027 | 1.75E-17 | -0.0018 | 0.0277 | 0.9478 |
| rs2173019 | 5 | 167614971 | A | T | 0.177 | 286 | 0.0282 | 0.0033 | 2.98E-17 | -0.0022 | 0.0345 | 0.9486 |
| rs10042827 | 5 | 170299916 | C | T | 0.681 | 150 | 0.0167 | 0.0027 | 9.41E-10 | 0.0334 | 0.0291 | 0.2522 |
| rs359431 | 5 | 173288534 | T | C | 0.560 | 122 | -0.0142 | 0.0026 | 3.16E-08 | -0.0007 | 0.0269 | 0.9806 |
| rs1150668 | 6 | 28129789 | G | T | 0.419 | 206 | -0.0185 | 0.0026 | 8.54E-13 | -0.0011 | 0.0268 | 0.9659 |
| rs1632941 | 6 | 29796685 | C | T | 0.460 | 153 | -0.0158 | 0.0026 | 6.67E-10 | -0.0090 | 0.0269 | 0.7375 |
| rs3218116 | 6 | 41901763 | T | C | 0.256 | 185 | -0.0198 | 0.0029 | 1.05E-11 | 0.0040 | 0.0306 | 0.8969 |
| rs160631 | 6 | 52895230 | G | T | 0.731 | 144 | -0.0173 | 0.0029 | 1.87E-09 | 0.0186 | 0.0304 | 0.5405 |
| rs7743165 | 6 | 67521222 | G | T | 0.495 | 228 | 0.0193 | 0.0025 | 4.15E-14 | 0.0147 | 0.0269 | 0.5857 |
| rs10945141 | 6 | 69470709 | A | G | 0.263 | 157 | 0.0181 | 0.0029 | 3.59E-10 | -0.0328 | 0.0306 | 0.2847 |
| rs17554906 | 6 | 92226609 | C | G | 0.444 | 122 | 0.0142 | 0.0026 | 3.14E-08 | 0.0256 | 0.0266 | 0.3357 |
| rs619087 | 6 | 94175279 | G | A | 0.422 | 122 | 0.0143 | 0.0026 | 3.1E-08 | -0.0060 | 0.0268 | 0.8215 |
| rs6568832 | 6 | 97702876 | A | G | 0.754 | 163 | 0.0189 | 0.0030 | 1.74E-10 | -0.0001 | 0.0311 | 0.9973 |
| rs12195240 | 6 | 98636905 | A | G | 0.285 | 312 | 0.0249 | 0.0028 | 1.08E-18 | 0.0161 | 0.0299 | 0.5905 |
| rs6936160 | 6 | 100347745 | T | C | 0.698 | 210 | 0.0201 | 0.0028 | 4.2E-13 | 0.0472 | 0.0290 | 0.1034 |
| rs3800227 | 6 | 108994161 | G | A | 0.742 | 139 | 0.0172 | 0.0029 | 3.64E-09 | 0.0114 | 0.0310 | 0.7141 |
| rs118202 | 6 | 111658371 | T | G | 0.812 | 508 | -0.0367 | 0.0033 | 1.9E-29 | 0.0049 | 0.0345 | 0.8876 |
| rs73008357 | 6 | 156431856 | C | A | 0.121 | 130 | -0.0223 | 0.0040 | 2.44E-08 | 0.0433 | 0.0408 | 0.2887 |
| rs9331343 | 6 | 157738258 | C | T | 0.568 | 121 | -0.0141 | 0.0026 | 3.9E-08 | -0.0468 | 0.0272 | 0.0852 |
| rs10698713 | 6 | 158882320 | A | G | 0.054 | 142 | -0.0335 | 0.0056 | 2.38E-09 | 0.1017 | 0.0560 | 0.0691 |
| rs1737329 | 6 | 163807748 | G | C | 0.742 | 137 | 0.0170 | 0.0029 | 5.08E-09 | 0.0080 | 0.0301 | 0.7911 |
| rs6948707 | 7 | 1870794 | G | T | 0.419 | 356 | 0.0243 | 0.0026 | 4.24E-21 | -0.0078 | 0.0268 | 0.7702 |
| rs13237637 | 7 | 3503207 | C | G | 0.485 | 345 | -0.0237 | 0.0025 | 1.54E-20 | -0.0209 | 0.0266 | 0.4313 |
| rs7809303 | 7 | 69484366 | A | G | 0.325 | 248 | -0.0214 | 0.0027 | 3.48E-15 | -0.0252 | 0.0285 | 0.3759 |
| rs7802996 | 7 | 77771983 | T | C | 0.166 | 149 | -0.0209 | 0.0034 | 1.06E-09 | -0.0267 | 0.0357 | 0.4541 |
| rs1030015 | 7 | 78139581 | T | G | 0.520 | 126 | 0.0143 | 0.0026 | 2.15E-08 | 0.0078 | 0.0266 | 0.7704 |
| rs4727189 | 7 | 88442568 | C | T | 0.344 | 123 | 0.0149 | 0.0027 | 0.00000003 | 0.0093 | 0.0285 | 0.7442 |
| rs76841737 | 7 | 91281409 | G | C | 0.103 | 122 | -0.0231 | 0.0042 | 3.26E-08 | -0.0395 | 0.0437 | 0.3669 |
| rs11768481 | 7 | 96629103 | A | C | 0.340 | 190 | -0.0186 | 0.0027 | 5.23E-12 | -0.0153 | 0.0283 | 0.5891 |
| rs1799068 | 7 | 97707069 | T | G | 0.379 | 160 | 0.0166 | 0.0026 | 2.59E-10 | -0.0254 | 0.0272 | 0.3508 |
| rs13437771 | 7 | 99071478 | G | A | 0.155 | 237 | -0.0271 | 0.0035 | 1.39E-14 | 0.0368 | 0.0371 | 0.3214 |
| rs11766326 | 7 | 111100585 | C | T | 0.506 | 190 | -0.0175 | 0.0026 | 1.79E-11 | -0.0069 | 0.0265 | 0.7949 |
| rs6968380 | 7 | 114940159 | A | G | 0.681 | 294 | -0.0234 | 0.0027 | 1.05E-17 | -0.0134 | 0.0288 | 0.6429 |
| rs10233018 | 7 | 117523709 | G | A | 0.516 | 373 | 0.0246 | 0.0025 | 4.77E-22 | 0.0011 | 0.0268 | 0.9679 |
| rs10953957 | 7 | 121954709 | A | G | 0.386 | 121 | 0.0144 | 0.0026 | 3.66E-08 | 0.0160 | 0.0275 | 0.5608 |
| rs77283305 | 7 | 132593831 | A | G | 0.306 | 121 | -0.0152 | 0.0028 | 3.91E-08 | 0.0585 | 0.0286 | 0.0407 |
| rs10279261 | 7 | 133589846 | A | G | 0.618 | 207 | -0.0189 | 0.0026 | 6.05E-13 | -0.0136 | 0.0276 | 0.6221 |
| rs4326350 | 8 | 10763655 | G | C | 0.493 | 191 | -0.0176 | 0.0026 | 5.16E-12 | -0.0330 | 0.0265 | 0.2142 |
| rs11783093 | 8 | 27425349 | T | C | 0.158 | 728 | -0.0471 | 0.0035 | 2.07E-41 | -0.0634 | 0.0368 | 0.0846 |
| rs7836565 | 8 | 52569449 | T | C | 0.718 | 120 | -0.0155 | 0.0028 | 4.36E-08 | 0.0434 | 0.0299 | 0.1469 |
| rs13261666 | 8 | 59814666 | T | G | 0.517 | 246 | -0.0200 | 0.0025 | 4.36E-15 | -0.0024 | 0.0268 | 0.9278 |
| rs3850736 | 8 | 64912021 | G | C | 0.474 | 225 | 0.0191 | 0.0026 | 6.43E-14 | 0.0200 | 0.0267 | 0.4541 |
| rs2063976 | 8 | 91096366 | T | C | 0.665 | 224 | -0.0202 | 0.0027 | 7.45E-14 | -0.0432 | 0.0282 | 0.1260 |
| rs6986430 | 8 | 93048104 | C | T | 0.222 | 252 | -0.0243 | 0.0031 | 1.99E-15 | 0.0046 | 0.0321 | 0.8851 |
| rs9987376 | 8 | 93190014 | G | T | 0.574 | 252 | -0.0205 | 0.0026 | 2.01E-15 | -0.0379 | 0.0267 | 0.1568 |
| rs290601 | 8 | 115374642 | T | C | 0.274 | 130 | 0.0163 | 0.0029 | 1.14E-08 | 0.0422 | 0.0298 | 0.1559 |
| rs3847244 | 9 | 3025368 | T | C | 0.470 | 214 | 0.0187 | 0.0026 | 2.6E-13 | -0.0455 | 0.0273 | 0.0956 |
| rs11791671 | 9 | 3398679 | T | C | 0.067 | 120 | 0.0279 | 0.0051 | 4.24E-08 | -0.0218 | 0.0531 | 0.6809 |
| rs7024924 | 9 | 8282399 | C | T | 0.174 | 126 | 0.0189 | 0.0034 | 1.9E-08 | -0.0011 | 0.0346 | 0.9745 |
| rs1931431 | 9 | 11161799 | C | G | 0.478 | 204 | 0.0182 | 0.0026 | 8.56E-13 | 0.0179 | 0.0267 | 0.5027 |
| rs7867822 | 9 | 20676454 | G | A | 0.673 | 124 | -0.0151 | 0.0027 | 2.76E-08 | -0.0249 | 0.0285 | 0.3839 |
| rs10966092 | 9 | 23831658 | C | T | 0.267 | 202 | -0.0205 | 0.0029 | 1.12E-12 | -0.0423 | 0.0309 | 0.1719 |
| rs10969352 | 9 | 29747488 | A | T | 0.500 | 127 | 0.0143 | 0.0025 | 1.82E-08 | 0.0066 | 0.0267 | 0.8046 |
| rs4877285 | 9 | 81354129 | A | G | 0.668 | 180 | -0.0181 | 0.0027 | 2.1E-11 | 0.0004 | 0.0286 | 0.9887 |
| rs1930371 | 9 | 81444104 | T | C | 0.241 | 134 | -0.0172 | 0.0030 | 7.09E-09 | -0.0027 | 0.0311 | 0.9308 |
| rs2378662 | 9 | 86707289 | A | G | 0.541 | 142 | 0.0152 | 0.0026 | 2.67E-09 | 0.0132 | 0.0269 | 0.6230 |
| rs1927901 | 9 | 120519111 | C | T | 0.553 | 122 | -0.0142 | 0.0026 | 3.1E-08 | 0.0131 | 0.0267 | 0.6236 |
| rs4837631 | 9 | 122061948 | T | C | 0.446 | 144 | -0.0154 | 0.0026 | 2.03E-09 | 0.0486 | 0.0266 | 0.0677 |
| rs1759433 | 9 | 128073097 | A | G | 0.480 | 145 | 0.0154 | 0.0026 | 1.69E-09 | 0.0094 | 0.0267 | 0.7237 |
| rs34553878 | 9 | 134334588 | G | A | 0.111 | 148 | 0.0247 | 0.0041 | 1.17E-09 | -0.0438 | 0.0426 | 0.3045 |
| rs7026534 | 9 | 134907263 | G | T | 0.704 | 142 | -0.0166 | 0.0028 | 2.68E-09 | -0.0496 | 0.0288 | 0.0845 |
| rs10858334 | 9 | 137989785 | G | C | 0.140 | 155 | 0.0229 | 0.0038 | 1.18E-09 | 0.0605 | 0.0372 | 0.1045 |
| rs10905461 | 10 | 8803551 | C | T | 0.748 | 125 | -0.0164 | 0.0029 | 2.36E-08 | 0.0349 | 0.0305 | 0.2529 |
| rs7920501 | 10 | 10043159 | A | T | 0.465 | 148 | -0.0155 | 0.0026 | 1.25E-09 | -0.0087 | 0.0268 | 0.7448 |
| rs1291821 | 10 | 11133823 | G | A | 0.534 | 129 | 0.0145 | 0.0026 | 1.39E-08 | 0.0539 | 0.0270 | 0.0460 |
| rs11258417 | 10 | 13533053 | T | C | 0.391 | 124 | -0.0145 | 0.0026 | 2.71E-08 | 0.0192 | 0.0272 | 0.4817 |
| rs7072776 | 10 | 22032942 | G | A | 0.712 | 244 | -0.0220 | 0.0028 | 5.66E-15 | 0.0289 | 0.0300 | 0.3353 |
| rs2796793 | 10 | 36634124 | A | G | 0.452 | 128 | 0.0145 | 0.0026 | 1.55E-08 | -0.0063 | 0.0267 | 0.8148 |
| rs1733760 | 10 | 56698174 | C | T | 0.510 | 134 | 0.0148 | 0.0025 | 6.7E-09 | -0.0113 | 0.0269 | 0.6754 |
| rs7921378 | 10 | 63674885 | C | G | 0.482 | 334 | -0.0233 | 0.0025 | 6.1E-20 | -0.0289 | 0.0268 | 0.2806 |
| rs11594623 | 10 | 103960351 | C | T | 0.234 | 333 | 0.0274 | 0.0030 | 7.45E-20 | -0.0045 | 0.0307 | 0.8836 |
| rs28408682 | 10 | 104403310 | G | A | 0.600 | 164 | 0.0167 | 0.0026 | 1.41E-10 | -0.0404 | 0.0269 | 0.1334 |
| rs12244388 | 10 | 104640052 | A | G | 0.350 | 374 | 0.0258 | 0.0027 | 4.31E-22 | -0.0704 | 0.0283 | 0.0128 |
| rs11192347 | 10 | 106929313 | A | G | 0.104 | 161 | -0.0265 | 0.0043 | 6.15E-10 | 0.0307 | 0.0426 | 0.4710 |
| rs10885480 | 10 | 115378364 | C | T | 0.284 | 175 | -0.0187 | 0.0028 | 3.83E-11 | 0.0485 | 0.0293 | 0.0979 |
| rs4752018 | 10 | 118678712 | A | C | 0.231 | 156 | 0.0189 | 0.0030 | 4.42E-10 | -0.0057 | 0.0319 | 0.8590 |
| rs9423279 | 10 | 125680419 | G | C | 0.645 | 195 | -0.0186 | 0.0027 | 3.06E-12 | 0.0063 | 0.0287 | 0.8260 |
| rs6265 | 11 | 27679916 | T | C | 0.188 | 322 | -0.0293 | 0.0033 | 2.81E-19 | -0.0141 | 0.0342 | 0.6796 |
| rs62618693 | 11 | 32956492 | T | C | 0.043 | 126 | -0.0353 | 0.0063 | 2.09E-08 | -0.0236 | 0.0663 | 0.7222 |
| rs2939756 | 11 | 41436297 | A | G | 0.480 | 152 | -0.0157 | 0.0026 | 7.45E-10 | -0.0040 | 0.0269 | 0.8818 |
| rs1381775 | 11 | 42442826 | C | T | 0.712 | 123 | -0.0156 | 0.0028 | 2.79E-08 | -0.0219 | 0.0297 | 0.4621 |
| rs2959084 | 11 | 46078656 | A | G | 0.705 | 150 | 0.0171 | 0.0028 | 9.82E-10 | 0.0046 | 0.0295 | 0.8766 |
| rs3740977 | 11 | 46393574 | C | T | 0.167 | 130 | 0.0195 | 0.0034 | 1.17E-08 | 0.0032 | 0.0353 | 0.9287 |
| rs61886926 | 11 | 64133552 | T | C | 0.384 | 188 | -0.0179 | 0.0026 | 7.3E-12 | 0.0450 | 0.0275 | 0.1018 |
| rs61884449 | 11 | 64485193 | T | C | 0.149 | 125 | 0.0200 | 0.0036 | 2.32E-08 | -0.0242 | 0.0374 | 0.5172 |
| rs644740 | 11 | 65561468 | T | C | 0.457 | 121 | -0.0141 | 0.0026 | 3.67E-08 | -0.0237 | 0.0269 | 0.3767 |
| rs7943721 | 11 | 73309393 | A | G | 0.829 | 157 | -0.0212 | 0.0034 | 3.58E-10 | 0.0058 | 0.0355 | 0.8704 |
| rs7929518 | 11 | 85980958 | G | A | 0.773 | 160 | 0.0192 | 0.0030 | 2.55E-10 | 0.0144 | 0.0324 | 0.6575 |
| rs586699 | 11 | 92289734 | A | G | 0.543 | 134 | -0.0148 | 0.0026 | 7.29E-09 | -0.0033 | 0.0268 | 0.9024 |
| rs76460663 | 11 | 111979741 | G | C | 0.041 | 174 | -0.0423 | 0.0064 | 4.15E-11 | 0.0445 | 0.0681 | 0.5135 |
| rs2155646 | 11 | 112912811 | C | T | 0.400 | 845 | 0.0378 | 0.0026 | 9.44E-48 | 0.0095 | 0.0273 | 0.7274 |
| rs1713676 | 11 | 113660576 | G | A | 0.523 | 172 | -0.0167 | 0.0026 | 5.38E-11 | 0.0275 | 0.0267 | 0.3042 |
| rs238896 | 11 | 113994505 | A | G | 0.490 | 175 | -0.0169 | 0.0025 | 3.65E-11 | -0.0278 | 0.0269 | 0.3016 |
| rs540860 | 11 | 121530888 | G | A | 0.543 | 190 | 0.0176 | 0.0026 | 5.75E-12 | 0.0087 | 0.0267 | 0.7432 |
| rs1106363 | 11 | 131966264 | T | C | 0.345 | 168 | 0.0174 | 0.0027 | 9.2E-11 | -0.0095 | 0.0283 | 0.7364 |
| rs2010921 | 11 | 132098205 | A | G | 0.311 | 160 | 0.0174 | 0.0028 | 2.47E-10 | 0.0508 | 0.0286 | 0.0758 |
| rs11057005 | 12 | 16748721 | G | A | 0.441 | 150 | -0.0157 | 0.0026 | 9.12E-10 | 0.0189 | 0.0271 | 0.4868 |
| rs13906 | 12 | 49952394 | T | C | 0.109 | 144 | -0.0245 | 0.0041 | 1.98E-09 | 0.0372 | 0.0439 | 0.3974 |
| rs4759229 | 12 | 56474480 | G | A | 0.656 | 135 | 0.0156 | 0.0027 | 6.53E-09 | -0.0066 | 0.0280 | 0.8131 |
| rs7969559 | 12 | 69655167 | G | A | 0.713 | 146 | -0.0170 | 0.0028 | 1.53E-09 | 0.0265 | 0.0296 | 0.3707 |
| rs7134009 | 12 | 75263193 | C | T | 0.287 | 126 | -0.0158 | 0.0029 | 4.3E-08 | -0.0239 | 0.0298 | 0.4217 |
| rs77215829 | 12 | 112618346 | C | A | 0.131 | 162 | -0.0240 | 0.0038 | 2.02E-10 | -0.0561 | 0.0403 | 0.1634 |
| rs1109480 | 12 | 121083279 | A | G | 0.384 | 162 | -0.0167 | 0.0026 | 1.84E-10 | -0.0070 | 0.0279 | 0.8013 |
| rs11611651 | 12 | 133380790 | A | G | 0.087 | 144 | 0.0271 | 0.0045 | 2.05E-09 | 0.0637 | 0.0461 | 0.1673 |
| rs17197663 | 13 | 38172867 | A | G | 0.125 | 126 | -0.0216 | 0.0039 | 2.06E-08 | -0.0096 | 0.0398 | 0.8086 |
| rs4264267 | 13 | 38359676 | T | C | 0.527 | 134 | 0.0148 | 0.0026 | 6.82E-09 | 0.0237 | 0.0270 | 0.3797 |
| rs61959481 | 13 | 55834929 | A | G | 0.210 | 169 | -0.0203 | 0.0031 | 7.95E-11 | -0.0620 | 0.0347 | 0.0743 |
| rs9538162 | 13 | 59265043 | C | T | 0.416 | 181 | 0.0174 | 0.0026 | 1.76E-11 | -0.0164 | 0.0269 | 0.5415 |
| rs55786907 | 13 | 59871584 | G | A | 0.162 | 127 | 0.0194 | 0.0035 | 1.84E-08 | -0.0252 | 0.0366 | 0.4917 |
| rs4886207 | 13 | 60705792 | C | T | 0.637 | 150 | -0.0162 | 0.0026 | 8.78E-10 | -0.0127 | 0.0277 | 0.6463 |
| rs9540731 | 13 | 66949370 | T | C | 0.509 | 194 | -0.0177 | 0.0025 | 3.42E-12 | -0.0074 | 0.0265 | 0.7818 |
| rs9545155 | 13 | 80191873 | C | T | 0.478 | 159 | -0.0161 | 0.0026 | 3.04E-10 | 0.0026 | 0.0266 | 0.9212 |
| rs1772572 | 13 | 81191176 | A | C | 0.324 | 154 | -0.0169 | 0.0027 | 5.62E-10 | -0.0278 | 0.0285 | 0.3291 |
| rs75674569 | 13 | 96823724 | A | G | 0.100 | 142 | -0.0253 | 0.0043 | 2.58E-09 | -0.0152 | 0.0445 | 0.7333 |
| rs7333559 | 13 | 100546450 | A | G | 0.783 | 226 | -0.0232 | 0.0031 | 5.94E-14 | 0.0163 | 0.0327 | 0.6191 |
| rs1108130 | 13 | 100648356 | A | T | 0.212 | 236 | 0.0239 | 0.0031 | 1.57E-14 | 0.0161 | 0.0331 | 0.6276 |
| rs12855717 | 13 | 101252635 | T | C | 0.538 | 148 | 0.0155 | 0.0026 | 1.22E-09 | 0.0217 | 0.0269 | 0.4199 |
| rs12878369 | 14 | 28346502 | A | C | 0.415 | 182 | 0.0174 | 0.0026 | 1.6E-11 | -0.0173 | 0.0272 | 0.5235 |
| rs9323328 | 14 | 58653514 | G | A | 0.537 | 124 | -0.0142 | 0.0026 | 2.55E-08 | -0.0147 | 0.0267 | 0.5807 |
| rs1811739 | 14 | 77529375 | A | G | 0.248 | 153 | 0.0183 | 0.0030 | 5.97E-10 | 0.0249 | 0.0309 | 0.4201 |
| rs8005334 | 14 | 79563654 | G | T | 0.360 | 158 | 0.0167 | 0.0027 | 3.44E-10 | -0.0201 | 0.0278 | 0.4696 |
| rs34940743 | 14 | 80102233 | G | A | 0.346 | 141 | 0.0159 | 0.0027 | 2.8E-09 | 0.0553 | 0.0277 | 0.0464 |
| rs2925128 | 14 | 98362355 | T | C | 0.385 | 165 | 0.0168 | 0.0027 | 3.67E-10 | -0.0350 | 0.0275 | 0.2032 |
| rs1381287 | 14 | 98597552 | T | C | 0.467 | 199 | 0.0180 | 0.0026 | 1.81E-12 | 0.0263 | 0.0269 | 0.3283 |
| rs55913542 | 14 | 99693843 | T | G | 0.175 | 123 | 0.0186 | 0.0034 | 3.25E-08 | 0.0369 | 0.0347 | 0.2863 |
| rs1435672 | 15 | 36399479 | C | T | 0.560 | 121 | 0.0141 | 0.0026 | 3.82E-08 | 0.0063 | 0.0270 | 0.8166 |
| rs281296 | 15 | 47685010 | A | G | 0.357 | 345 | 0.0247 | 0.0027 | 1.59E-20 | 0.0202 | 0.0278 | 0.4691 |
| rs56902655 | 15 | 63898709 | G | T | 0.136 | 138 | -0.0219 | 0.0037 | 4.09E-09 | -0.0381 | 0.0387 | 0.3247 |
| rs2289791 | 15 | 67476952 | T | G | 0.247 | 144 | -0.0177 | 0.0030 | 2.01E-09 | 0.0009 | 0.0311 | 0.9767 |
| rs60833441 | 15 | 74048768 | G | A | 0.461 | 125 | -0.0143 | 0.0026 | 2.28E-08 | -0.0408 | 0.0269 | 0.1290 |
| rs62007780 | 15 | 78025464 | T | G | 0.416 | 152 | -0.0159 | 0.0026 | 7.48E-10 | -0.0482 | 0.0267 | 0.0709 |
| rs4310804 | 15 | 96858409 | G | C | 0.247 | 152 | -0.0182 | 0.0030 | 7.55E-10 | -0.0182 | 0.0313 | 0.5613 |
| rs8027457 | 15 | 99204101 | C | T | 0.511 | 144 | 0.0153 | 0.0025 | 1.88E-09 | 0.0293 | 0.0266 | 0.2701 |
| rs1139897 | 16 | 720986 | A | G | 0.230 | 253 | -0.0241 | 0.0030 | 1.77E-15 | -0.0286 | 0.0319 | 0.3701 |
| rs11076962 | 16 | 5811367 | C | T | 0.279 | 166 | 0.0183 | 0.0028 | 1.2E-10 | 0.0331 | 0.0294 | 0.2605 |
| rs7192140 | 16 | 10173748 | C | T | 0.498 | 176 | -0.0169 | 0.0025 | 3.4E-11 | 0.0194 | 0.0269 | 0.4709 |
| rs9922607 | 16 | 17570220 | T | C | 0.200 | 194 | -0.0222 | 0.0032 | 3.42E-12 | -0.0113 | 0.0329 | 0.7305 |
| rs9941217 | 16 | 18050926 | G | C | 0.352 | 194 | -0.0186 | 0.0027 | 3.5E-12 | -0.0412 | 0.0279 | 0.1396 |
| rs7188873 | 16 | 24727064 | G | A | 0.613 | 241 | 0.0203 | 0.0026 | 8.46E-15 | 0.0016 | 0.0276 | 0.9549 |
| rs6497840 | 16 | 25351633 | A | G | 0.707 | 265 | 0.0228 | 0.0029 | 2.01E-15 | 0.0011 | 0.0297 | 0.9706 |
| rs4785187 | 16 | 49766772 | A | G | 0.223 | 170 | 0.0200 | 0.0031 | 6.55E-11 | 0.0151 | 0.0322 | 0.6398 |
| rs8050598 | 16 | 49891964 | T | C | 0.254 | 163 | 0.0187 | 0.0029 | 1.76E-10 | -0.0221 | 0.0316 | 0.4850 |
| rs12918191 | 16 | 50945156 | G | A | 0.243 | 176 | -0.0197 | 0.0030 | 3.14E-11 | -0.0104 | 0.0311 | 0.7382 |
| rs9302604 | 16 | 69576894 | G | A | 0.435 | 212 | 0.0187 | 0.0026 | 3.29E-13 | 0.0251 | 0.0269 | 0.3510 |
| rs62052916 | 16 | 72574550 | T | A | 0.070 | 164 | -0.0319 | 0.0050 | 1.62E-10 | -0.0053 | 0.0507 | 0.9167 |
| rs4788676 | 16 | 72950468 | C | T | 0.229 | 137 | -0.0177 | 0.0030 | 4.92E-09 | -0.0094 | 0.0316 | 0.7654 |
| rs117657830 | 16 | 75766873 | G | A | 0.042 | 140 | -0.0378 | 0.0064 | 3.18E-09 | 0.0282 | 0.0627 | 0.6529 |
| rs1050847 | 16 | 87443734 | T | C | 0.559 | 134 | -0.0148 | 0.0026 | 7.37E-09 | -0.0130 | 0.0270 | 0.6308 |
| rs11642231 | 16 | 89608702 | A | G | 0.369 | 140 | -0.0156 | 0.0026 | 3.44E-09 | 0.0371 | 0.0273 | 0.1742 |
| rs4790874 | 17 | 1995177 | T | C | 0.532 | 187 | 0.0174 | 0.0026 | 8.43E-12 | -0.0034 | 0.0266 | 0.8992 |
| rs28441558 | 17 | 7803118 | C | T | 0.056 | 166 | -0.0356 | 0.0055 | 1.24E-10 | 0.0180 | 0.0561 | 0.7480 |
| rs11651955 | 17 | 16235462 | A | G | 0.499 | 121 | -0.0140 | 0.0025 | 3.74E-08 | 0.0203 | 0.0266 | 0.4454 |
| rs67777803 | 17 | 27323322 | T | G | 0.172 | 212 | -0.0246 | 0.0034 | 3.18E-13 | -0.0343 | 0.0354 | 0.3322 |
| rs2344976 | 17 | 30685935 | C | T | 0.612 | 133 | -0.0151 | 0.0026 | 7.98E-09 | 0.0085 | 0.0274 | 0.7558 |
| rs17692129 | 17 | 44793283 | T | C | 0.331 | 210 | 0.0196 | 0.0027 | 4.57E-13 | 0.0344 | 0.0281 | 0.2214 |
| rs75919030 | 17 | 50193197 | C | T | 0.267 | 212 | -0.0210 | 0.0029 | 3.35E-13 | 0.0334 | 0.0302 | 0.2685 |
| rs2587507 | 17 | 77790135 | C | T | 0.502 | 132 | -0.0147 | 0.0025 | 8.69E-09 | -0.0149 | 0.0265 | 0.5733 |
| rs34342129 | 18 | 5872472 | C | T | 0.509 | 126 | -0.0143 | 0.0025 | 2.13E-08 | -0.0217 | 0.0267 | 0.4156 |
| rs4476253 | 18 | 25253297 | A | G | 0.240 | 154 | -0.0185 | 0.0030 | 5.78E-10 | -0.0666 | 0.0318 | 0.0359 |
| rs7505855 | 18 | 31696075 | T | C | 0.586 | 172 | -0.0170 | 0.0026 | 5.31E-11 | -0.0348 | 0.0269 | 0.1965 |
| rs8096225 | 18 | 36921851 | C | A | 0.703 | 124 | 0.0155 | 0.0028 | 2.63E-08 | 0.0057 | 0.0289 | 0.8431 |
| rs67050670 | 18 | 39297254 | G | A | 0.229 | 179 | -0.0203 | 0.0030 | 2.34E-11 | -0.0558 | 0.0322 | 0.0833 |
| rs72898831 | 18 | 42658643 | G | A | 0.155 | 192 | -0.0244 | 0.0035 | 4.14E-12 | 0.0342 | 0.0362 | 0.3446 |
| rs1373178 | 18 | 49967811 | G | T | 0.588 | 246 | -0.0203 | 0.0026 | 4.16E-15 | -0.0162 | 0.0272 | 0.5517 |
| rs62098013 | 18 | 50863861 | A | G | 0.365 | 179 | 0.0177 | 0.0026 | 2.24E-11 | 0.0294 | 0.0280 | 0.2947 |
| rs72938304 | 18 | 53661743 | A | G | 0.113 | 183 | -0.0272 | 0.0040 | 1.36E-11 | -0.0257 | 0.0411 | 0.5323 |
| rs11872397 | 18 | 72535282 | A | G | 0.253 | 136 | -0.0171 | 0.0029 | 5.2E-09 | -0.0078 | 0.0306 | 0.7992 |
| rs71367544 | 18 | 77574374 | T | C | 0.203 | 168 | 0.0206 | 0.0032 | 8.54E-11 | 0.0572 | 0.0322 | 0.0759 |
| rs76608582 | 19 | 4474725 | A | C | 0.049 | 137 | -0.0345 | 0.0059 | 4.88E-09 | -0.0930 | 0.0669 | 0.1644 |
| rs10853981 | 19 | 4965064 | A | G | 0.330 | 119 | 0.0148 | 0.0027 | 4.88E-08 | 0.0328 | 0.0282 | 0.2446 |
| rs113230003 | 19 | 18460956 | A | G | 0.255 | 167 | -0.0189 | 0.0029 | 1.05E-10 | -0.0473 | 0.0310 | 0.1275 |
| rs8103660 | 19 | 18566395 | C | T | 0.354 | 141 | 0.0158 | 0.0027 | 3.03E-09 | 0.0290 | 0.0277 | 0.2955 |
| rs117734003 | 19 | 51129745 | C | G | 0.067 | 142 | 0.0303 | 0.0051 | 2.57E-09 | 0.0032 | 0.0532 | 0.9527 |
| rs1126757 | 19 | 55879872 | T | C | 0.473 | 123 | 0.0142 | 0.0026 | 2.92E-08 | 0.0237 | 0.0269 | 0.3795 |
| rs6050446 | 20 | 25195509 | G | A | 0.971 | 205 | 0.0544 | 0.0076 | 8.8E-13 | 0.0437 | 0.0791 | 0.5804 |
| rs6073075 | 20 | 42015801 | A | T | 0.824 | 125 | -0.0187 | 0.0034 | 2.44E-08 | 0.0249 | 0.0354 | 0.4823 |
| rs910912 | 20 | 54462393 | C | T | 0.739 | 134 | -0.0168 | 0.0029 | 7.82E-09 | 0.0002 | 0.0300 | 0.9934 |
| rs6011779 | 20 | 61984317 | T | C | 0.806 | 142 | -0.0192 | 0.0032 | 2.83E-09 | -0.0031 | 0.0340 | 0.9284 |
| rs3810496 | 20 | 62406886 | C | T | 0.619 | 147 | 0.0159 | 0.0026 | 1.54E-09 | 0.0211 | 0.0277 | 0.4462 |
| rs4818005 | 21 | 40588819 | A | G | 0.581 | 250 | -0.0204 | 0.0026 | 1.09E-14 | -0.0353 | 0.0270 | 0.1915 |
| rs4822102 | 22 | 42698430 | T | C | 0.618 | 159 | -0.0165 | 0.0026 | 2.78E-10 | 0.0070 | 0.0273 | 0.7966 |
| rs9627272 | 22 | 46442288 | C | G | 0.407 | 142 | -0.0155 | 0.0026 | 2.42E-09 | 0.0024 | 0.0273 | 0.9315 |

SNP indicates single-nucleotide polymorphism; Chr, chromosome; Pos, position; EA, effect allele; OA, other allele; EAF, effect allele frequency; F, F-statistics; SmkInit, smoking initiation; CAD, coronary artery disease; SE, standard error.

**Table S2.** Characteristics of SNPs and their associations with AgeSmk and CAD in patients with diabetes.

| SNP | Chr | Pos | EA | OA | EAF | F | AgeSmk | | | CAD | | |
| --- | --- | --- | --- | --- | --- | --- | --- | --- | --- | --- | --- | --- |
|  |  |  |  |  |  |  | Beta | SE | P | Beta | SE | P |
| rs72853300 | 2 | 145638766 | T | C | 0.153 | 32 | 0.0190 | 0.0034 | 1.75E-08 | -0.0429 | 0.0367 | 0.2421 |
| rs7559982 | 2 | 63622309 | A | T | 0.564 | 50 | -0.0172 | 0.0024 | 1.67E-12 | 0.0002 | 0.0270 | 0.9939 |
| rs11915747 | 3 | 85699040 | G | C | 0.354 | 64 | 0.0202 | 0.0025 | 1.57E-15 | -0.0480 | 0.0280 | 0.0867 |
| rs13136239 | 4 | 140908755 | A | G | 0.342 | 34 | 0.0148 | 0.0026 | 6.29E-09 | 0.0164 | 0.0281 | 0.5585 |
| rs2471711 | 4 | 28589079 | T | C | 0.152 | 33 | -0.0192 | 0.0034 | 1.19E-08 | 0.0105 | 0.0359 | 0.7699 |
| rs624833 | 4 | 2881256 | G | T | 0.302 | 36 | 0.0157 | 0.0026 | 2.36E-09 | -0.0421 | 0.0291 | 0.1476 |
| rs7682598 | 4 | 68000888 | G | A | 0.771 | 36 | 0.0173 | 0.0029 | 2.09E-09 | -0.0669 | 0.0315 | 0.0335 |
| rs1403174 | 7 | 2032865 | T | A | 0.579 | 40 | 0.0155 | 0.0025 | 2.50E-10 | -0.0017 | 0.0268 | 0.9491 |
| rs11780471 | 8 | 27344719 | A | G | 0.060 | 42 | 0.0330 | 0.0051 | 9.44E-11 | -0.0926 | 0.0544 | 0.0890 |

SNP indicates single-nucleotide polymorphism; Chr, chromosome; Pos, position; EA, effect allele; OA, other allele; EAF, effect allele frequency; F, F-statistics; AgeSmk, age at initiation of regular smoking; CAD, coronary artery disease; SE, standard error.

**Table S3.** Characteristics of SNPs and their associations with CigDay and CAD in patients with diabetes.

| SNP | Chr | Pos | EA | OA | EAF | F | CigDay | | | CAD | | |
| --- | --- | --- | --- | --- | --- | --- | --- | --- | --- | --- | --- | --- |
|  |  |  |  |  |  |  | Beta | SE | P | Beta | SE | P |
| rs11264100 | 1 | 35591626 | G | A | 0.876 | 36 | -0.0222 | 0.0037 | 2.22E-09 | -0.0736 | 0.0433 | 0.0891 |
| rs2072659 | 1 | 154548521 | G | C | 0.099 | 54 | -0.0300 | 0.0041 | 2.51E-13 | 0.0513 | 0.0456 | 0.2612 |
| rs34973462 | 1 | 175993820 | T | C | 0.334 | 34 | 0.0151 | 0.0026 | 5.85E-09 | 0.0245 | 0.0280 | 0.3826 |
| rs7599488 | 2 | 60718347 | T | C | 0.437 | 33 | 0.0141 | 0.0025 | 8.95E-09 | 0.0391 | 0.0268 | 0.1441 |
| rs78408772 | 2 | 62710608 | T | C | 0.102 | 30 | -0.0220 | 0.0040 | 4.51E-08 | -0.0098 | 0.0446 | 0.8259 |
| rs10204824 | 2 | 148372720 | G | A | 0.639 | 50 | -0.0180 | 0.0025 | 1.35E-12 | -0.0032 | 0.0284 | 0.9096 |
| rs2084533 | 3 | 16872929 | T | C | 0.321 | 38 | 0.0161 | 0.0026 | 6.53E-10 | 0.0044 | 0.0290 | 0.8792 |
| rs7431710 | 3 | 48935583 | A | G | 0.654 | 51 | -0.0183 | 0.0026 | 1.04E-12 | -0.0547 | 0.0279 | 0.0502 |
| rs699165 | 3 | 136224697 | G | A | 0.745 | 33 | 0.0161 | 0.0028 | 8.09E-09 | 0.0243 | 0.0306 | 0.4266 |
| rs28813180 | 3 | 158083918 | A | G | 0.498 | 41 | -0.0155 | 0.0024 | 1.95E-10 | 0.0364 | 0.0264 | 0.1678 |
| rs1024323 | 4 | 3006043 | T | C | 0.382 | 33 | -0.0144 | 0.0025 | 8.66E-09 | -0.0418 | 0.0276 | 0.1292 |
| rs11940255 | 4 | 67086288 | A | G | 0.717 | 40 | -0.0172 | 0.0027 | 2.20E-10 | -0.0257 | 0.0301 | 0.3936 |
| rs10454798 | 4 | 67980830 | T | G | 0.253 | 32 | 0.0158 | 0.0028 | 1.53E-08 | 0.0347 | 0.0306 | 0.2565 |
| rs7766641 | 6 | 26184102 | A | G | 0.272 | 40 | -0.0173 | 0.0027 | 2.91E-10 | -0.0005 | 0.0310 | 0.9878 |
| rs215600 | 7 | 32333642 | A | G | 0.645 | 89 | -0.0240 | 0.0025 | 4.02E-21 | -0.0227 | 0.0279 | 0.4152 |
| rs62447179 | 7 | 50339609 | A | G | 0.298 | 33 | -0.0153 | 0.0027 | 9.68E-09 | 0.0333 | 0.0287 | 0.2457 |
| rs73229090 | 8 | 27442127 | A | C | 0.112 | 46 | 0.0262 | 0.0039 | 1.14E-11 | -0.0363 | 0.0418 | 0.3849 |
| rs4236926 | 8 | 42578059 | G | T | 0.766 | 142 | 0.0343 | 0.0029 | 7.66E-33 | 0.0129 | 0.0321 | 0.6866 |
| rs790564 | 8 | 64604218 | C | A | 0.729 | 41 | -0.0176 | 0.0027 | 1.24E-10 | 0.0250 | 0.0300 | 0.4063 |
| rs75596189 | 9 | 136468701 | T | C | 0.112 | 86 | 0.0358 | 0.0039 | 1.84E-20 | 0.0512 | 0.0422 | 0.2257 |
| rs3025383 | 9 | 136502369 | C | T | 0.187 | 101 | -0.0314 | 0.0031 | 9.78E-24 | 0.0154 | 0.0345 | 0.6554 |
| rs7951365 | 11 | 16377044 | C | T | 0.310 | 46 | 0.0178 | 0.0026 | 1.53E-11 | -0.0353 | 0.0291 | 0.2250 |
| rs10742683 | 11 | 43667625 | A | G | 0.415 | 30 | -0.0135 | 0.0025 | 4.83E-08 | 0.0380 | 0.0269 | 0.1577 |
| rs113001570 | 11 | 46737412 | T | A | 0.067 | 37 | 0.0298 | 0.0049 | 1.04E-09 | -0.0137 | 0.0532 | 0.7977 |
| rs7125588 | 11 | 113436072 | G | A | 0.429 | 47 | -0.0169 | 0.0025 | 6.50E-12 | 0.0229 | 0.0269 | 0.3943 |
| rs11846838 | 14 | 104184737 | A | G | 0.327 | 34 | 0.0152 | 0.0026 | 5.03E-09 | 0.0079 | 0.0285 | 0.7823 |
| rs632811 | 15 | 59155050 | G | A | 0.330 | 47 | -0.0178 | 0.0028 | 1.67E-10 | 0.0024 | 0.0284 | 0.9334 |
| rs10519203 | 15 | 78814046 | A | G | 0.655 | 1342 | -0.0936 | 0.0026 | 3.12E-286 | 0.0389 | 0.0284 | 0.1704 |
| rs182317 | 15 | 89943601 | T | G | 0.355 | 38 | -0.0156 | 0.0026 | 1.31E-09 | 0.0020 | 0.0277 | 0.9412 |
| rs1592485 | 16 | 52093549 | A | C | 0.611 | 42 | -0.0162 | 0.0025 | 1.11E-10 | 0.0279 | 0.0276 | 0.3116 |
| rs12924872 | 16 | 69552215 | T | C | 0.463 | 30 | -0.0134 | 0.0024 | 4.39E-08 | -0.0171 | 0.0272 | 0.5309 |
| rs258321 | 16 | 89756473 | G | A | 0.429 | 41 | 0.0158 | 0.0025 | 1.53E-10 | -0.0100 | 0.0271 | 0.7118 |
| rs4144686 | 18 | 53251725 | A | G | 0.167 | 32 | -0.0186 | 0.0033 | 1.35E-08 | -0.0149 | 0.0354 | 0.6739 |
| rs4485470 | 18 | 62125063 | A | G | 0.592 | 38 | -0.0153 | 0.0025 | 7.05E-10 | 0.0106 | 0.0271 | 0.6954 |
| rs59208569 | 19 | 4044424 | C | G | 0.829 | 40 | 0.0205 | 0.0032 | 2.45E-10 | 0.0823 | 0.0371 | 0.0266 |
| rs56113850 | 19 | 41353107 | C | T | 0.555 | 457 | 0.0523 | 0.0025 | 4.01E-99 | -0.0027 | 0.0271 | 0.9193 |
| rs6078373 | 20 | 11863500 | A | G | 0.402 | 42 | 0.0161 | 0.0025 | 9.40E-11 | 0.0160 | 0.0272 | 0.5576 |
| rs1737894 | 20 | 31054702 | G | C | 0.408 | 46 | 0.0169 | 0.0025 | 9.90E-12 | 0.0311 | 0.0278 | 0.2621 |
| rs2273500 | 20 | 61986949 | C | T | 0.147 | 112 | 0.0364 | 0.0034 | 3.49E-26 | 0.0100 | 0.0379 | 0.7916 |
| rs7281463 | 21 | 40520783 | C | A | 0.413 | 31 | 0.0137 | 0.0025 | 3.15E-08 | 0.0340 | 0.0272 | 0.2112 |

SNP indicates single-nucleotide polymorphism; Chr, chromosome; Pos, position; EA, effect allele; OA, other allele; EAF, effect allele frequency; F, F-statistics; CigDay, cigarettes per day; CAD, coronary artery disease; SE, standard error.

**Table S4.** Characteristics of SNPs and their associations with SmkCes and CAD in patients with diabetes.

| SNP | Chr | Pos | EA | OA | EAF | F | SmkCes | | | CAD | | |
| --- | --- | --- | --- | --- | --- | --- | --- | --- | --- | --- | --- | --- |
|  |  |  |  |  |  |  | Beta | SE | P | Beta | SE | P |
| rs112187834 | 2 | 23953454 | A | T | 0.140 | 147 | 0.0334 | 0.0056 | 2.81E-09 | -0.0280 | 0.0380 | 0.4607 |
| rs7617480 | 3 | 49210732 | C | A | 0.773 | 208 | -0.0329 | 0.0047 | 1.68E-12 | -0.0243 | 0.0318 | 0.4458 |
| rs12203592 | 6 | 396321 | T | C | 0.176 | 135 | -0.0292 | 0.0051 | 1.21E-08 | 0.0037 | 0.0329 | 0.9110 |
| rs707968 | 6 | 35058117 | G | A | 0.681 | 129 | 0.0233 | 0.0042 | 2.76E-08 | 0.0517 | 0.0285 | 0.0694 |
| rs7778443 | 7 | 32314690 | C | T | 0.618 | 137 | -0.0230 | 0.0040 | 1.04E-08 | -0.0221 | 0.0274 | 0.4211 |
| rs1565735 | 8 | 27426077 | A | T | 0.199 | 208 | -0.0346 | 0.0049 | 1.54E-12 | -0.0504 | 0.0336 | 0.1338 |
| rs60749569 | 8 | 42602668 | T | A | 0.080 | 129 | -0.0401 | 0.0072 | 2.68E-08 | -0.0124 | 0.0474 | 0.7932 |
| rs9409844 | 9 | 136461851 | A | G | 0.045 | 162 | -0.0586 | 0.0094 | 4.37E-10 | 0.0842 | 0.0683 | 0.2178 |
| rs3025327 | 9 | 136467344 | C | G | 0.107 | 647 | 0.0786 | 0.0063 | 1.19E-35 | 0.0515 | 0.0423 | 0.2228 |
| rs1611124 | 9 | 136509275 | T | G | 0.068 | 142 | -0.0453 | 0.0078 | 5.26E-09 | 0.0258 | 0.0513 | 0.6152 |
| rs7109376 | 11 | 16372431 | A | T | 0.279 | 173 | 0.0281 | 0.0044 | 1.14E-10 | -0.0285 | 0.0301 | 0.3424 |
| rs591143 | 15 | 47647755 | T | C | 0.592 | 157 | -0.0243 | 0.0040 | 1.14E-09 | 0.0057 | 0.0271 | 0.8327 |
| rs3866543 | 15 | 76629609 | G | T | 0.523 | 135 | 0.0222 | 0.0039 | 1.35E-08 | -0.0133 | 0.0266 | 0.6163 |
| rs518425 | 15 | 78883813 | G | A | 0.285 | 208 | -0.0305 | 0.0043 | 1.72E-12 | -0.0346 | 0.0297 | 0.2432 |
| rs56113850 | 19 | 41353107 | C | T | 0.567 | 893 | -0.0576 | 0.0039 | 1.61E-48 | -0.0027 | 0.0271 | 0.9193 |
| rs6011779 | 20 | 61984317 | T | C | 0.806 | 429 | -0.0500 | 0.0050 | 9.89E-24 | -0.0031 | 0.0340 | 0.9284 |
| rs6089904 | 20 | 62018289 | T | A | 0.047 | 204 | -0.0642 | 0.0093 | 4.01E-12 | -0.0080 | 0.0644 | 0.9012 |
| rs9607805 | 22 | 41854446 | T | C | 0.725 | 190 | 0.0295 | 0.0044 | 1.37E-11 | -0.0058 | 0.0298 | 0.8451 |

SNP indicates single-nucleotide polymorphism; Chr, chromosome; Pos, position; EA, effect allele; OA, other allele; EAF, effect allele frequency; F, F-statistics; SmkCes, smoking cessation; CAD, coronary artery disease; SE, standard error.

**Table S5.** Power calculation for the Mendelian randomization analyses.

| Exposure | Outcome | Sample size | α | K | OR | R^2^ | Power |
| --- | --- | --- | --- | --- | --- | --- | --- |
| SmkInit | CAD in diabetes | 15,666 | 0.05 | 0.253 | 1.322 | 0.0468 | 0.94 |
| AgeSmk | CAD in diabetes | 15,666 | 0.05 | 0.253 | 0.214 | 0.0011 | 0.50 |
| CigDay | CAD in diabetes | 15,666 | 0.05 | 0.253 | 1.012 | 0.0107 | 0.05 |
| SmkCes | CAD in diabetes | 15,666 | 0.05 | 0.253 | 1.229 | 0.0081 | 0.19 |

SmkInit, smoking initiation; AgeSmk, age at initiation of regular smoking; CigDay, cigarettes per day; SmkCes, smoking cessation; CAD, coronary artery disease; α, type-I error rate; K, proportion of cases in the study; OR, True odds ratio of the outcome variable per standard deviation of the exposure variable; R^2^, proportion of variance explained for the association between the SNP the exposure variable.

**Figure S1.** Funnel plots of the Mendelian randomization estimate for the associations between smoking traits and coronary artery disease in patients with diabetes. A. funnel plot for smoking initiation; B. funnel plot for age at initiation of regular smoking; C. funnel plot for cigarette per day; D. funnel plot for smoking cessation. X axis presents the estimate and Y axis presents the relevant inverse standard error. The dots indicate each single-nucleotide polymorphism, and the line indicates the overall estimate using the inverse-variance-weighted method.
